# Clinical impact of the disposable ventouse iCup® versus a metallic vacuum cup: a multicenter randomized controlled trial

**DOI:** 10.1186/s12884-015-0771-1

**Published:** 2015-12-15

**Authors:** Véronique Equy, Sandra David-Tchouda, Michel Dreyfus, Didier Riethmuller, Françoise Vendittelli, Victoire Cabaud, Bruno Langer, Jennifer Margier, Jean-Luc Bosson, Jean-Patrick Schaal

**Affiliations:** Obstetrical Care Unit, ‘Hôpital Couple Enfant’, Grenoble University Hospital, CS 10217, 38043 Grenoble cedex 09, France; Grenoble University Hospital, Clinical and Medico-economic Evaluation Unit – Clinical Research and Innovation Department, Grenoble University Hospital, 38000 Grenoble, France; TIMC-Imag UMR 5525, Université Grenoble Alpes, 38000 Grenoble, France; Obstetrical Care Unit, ‘Hôpital Femme Enfant’, Caen University Hospital, 14033 Caen, France; Obstetrical Care Unit, ‘Pôle Mère-Femme’, Besançon University Hospital, 25030 Besançon, France; Obstetrical Care Unit, Site Estaing, Clermont-Ferrand University Hospital, 1 place Lucie et Raymond Aubrac, 63003 Clermont-Ferrand Cedex 1, France; Université d’Auvergne, EA 4681, PEPRADE (Périnatalité, grossesse, Environnement, PRAtiques médicales et DEveloppement), Clermont University, Université d’Auvergne, 63001 Clermont-Ferrand, France; Obstetrical Care Unit, Chambéry Hospital, 73011 Chambéry, France; Obstetrical Care Unit, ‘Hôpital Hautepierre’, Strasbourg University Hospital, 67098 Strasbourg, France; INSERM CIC 1406, Grenoble University Hospital, 38000 Grenoble, France

**Keywords:** Vacuum extraction, Delivery, Obstetrical, Randomized controlled trial

## Abstract

**Background:**

Assisted vaginal delivery by vacuum extraction is frequent. Metallic resterilizible metallic vacuum cups have been routinely used in France. In the last few years a new disposable semi-soft vacuum extraction cup, the iCup, has been introduced. Our objective was to compare maternal and new-born outcomes between this disposable cup and the commonly used Drapier-Faure metallic cup.

**Methods:**

This was a multicenter prospective randomized controlled open clinical trial performed in the maternity units of five university hospitals and one community hospital in France from October 2009 to February 2013. We included consecutive eligible women with a singleton gestation of at least 37 weeks who required vacuum assisted delivery. Women were randomized to **v**acuum extraction using the iCup or usual Drapier-Faure metallic cup. The primary outcome was a composite criterion including both the risk of cup dysfunction and the most frequent maternal and neonatal harms: the use of other instruments after attempted vacuum extraction, caesarean section after attempted vacuum extraction, three detachments of the cup, caput succedaneum, cephalohaematoma, episiotomy and perineal tears.

**Results:**

335 women were randomized to the disposable cup and 333 to extraction using the metallic cup. There was no significant difference between the two groups for the primary outcome. However, failed instrumental delivery was more frequent in the disposable cup group, mainly due to detachment: 35.6 % vs 7.1 %, *p <* 0.0001. Conversely, perineal tears were more frequent in the metallic cup group, especially third or fourth grade perineal tears: 1.7 % versus 5.0 %, *p =* 0.003. There were no significant differences between the two groups concerning post-partum haemorrhage, transfer to a neonatal intensive care unit (NICU) or serious adverse events.

**Conclusions:**

While the disposable cup had more detachments and extraction failures than the standard metallic cup, this innovative disposable device had the advantage of fewer perineal injuries.

**Trial registration:**

www.clinicaltrials.gov: NCT01058200 on Jan. 27 2010.

**Electronic supplementary material:**

The online version of this article (doi:10.1186/s12884-015-0771-1) contains supplementary material, which is available to authorized users.

## Background

Instrumental vaginal delivery is common practice worldwide for both maternal and foetal indications, and the instrument chosen depends on the indication, the pros and cons of each instrument and the choice of the operator. Currently, instrumental extraction is used in about 16 % of the 860 000 annual deliveries in France and ventouse extraction represents 40 % of assisted vaginal deliveries [1]. More recent data from the same registry described: 13.1 % of instrumental deliveries, of which 38.3 % were vacuum cup extraction (http://www.audipog.net/). While the use of forceps is accompanied by fewer extraction failures, the use of a ventouse is associated with less maternal or neonatal trauma [2–4].

In France the most frequently used metallic cup is the Drapier-Faure MiniCup® (Collin-Gentile-Drapier, Paris, France). It has a rigid cup with a vacuum aspiration system independent of the traction system. The disadvantage of this cup is that the lateral insertion of aspiration can lead to perineal wounds or vaginal injuries [5]. There are two main types of non-metallic vacuum cups, silicon cups and disposable cups. Silicon cups are sterilisable and resemble “trumpets” with a stem connected to the vacuum pump, which also serves for traction. The disposable Kiwi OmniCup® (Clinical Innovations, Heathrow, UK) ventouse has a rigid plastic cup with an integrated hand-held vacuum connection that allows both aspiration and traction [6–8]. The new iCup® vacuum cup is disposable and has a 5 cm diameter cup with a central traction strap and a flexible suction tube leading to an electric pump. The cup is made from a rigid plastic material, but the part of the cup in contact with the foetal scalp is made of polyurethane, a soft plastic material with adherent properties (Gyneas, Goussainville, France).

A Cochrane literature review on the choice of instruments for assisted vaginal delivery found only 10 randomized trials (1558 women) comparing soft with metallic vacuum cups among 32 trials worldwide [3]. These trials were run in 8 countries, mainly in Europe, Asia and to a lesser extent Africa, where vacuum cups are regularly used. The meta-analysis showed that extraction failures occurred more often with soft cups: RR 1.63 [1.17; 2.28], but that scalp injuries and cephalohaematomas were significantly less frequent: RR = 0.67 [95 % CI: 0.53; 0.86] and RR = 0.61 [95 % CI: 0.39; 0.95] respectively. No difference in maternal complications was found, in particular the rate of caesarean section, episiotomy, and perineal trauma [3]. However, the major drawback of most of the studies comparing metallic cups and soft cups was their heterogeneous control groups including different types of cups, which could bias the final results and/or they were single centre studies [3].

Finally, no study has been done with the recently introduced iCup® ventouse, an innovative compromise between metallic and soft cups. Thus, we aimed to compare this new disposable device with the metallic cup considered as the ‘gold-standard’ in France: the Drapier-Faure® vacuum cup, both in terms of risk of cup dysfunction and the most frequent maternal and neonatal harms.

## Methods

### Study population

The population of our study was women giving birth after 37 weeks of gestation requiring and able to have a vacuum assisted extraction.

Women were included if they were aged between 18 and 45 years, with a singleton pregnancy, cephalic presentation at delivery and if instrumental extraction was indicated.

Women, who refused to participate or were deprived of liberty by judicial or administrative decision or under legal protection, were not included.

### Study design

This multicentre prospective randomized controlled open superiority clinical trial followed the CONSORT statement of Non-Pharmacologic Treatment Interventions (http://www.consort-statement.org/checklists/view/648-non-pharmacologic-treatment/652-title) and was conducted from October 2009 to February 2013 in 6 maternity units in France (5 university hospitals and 1 community hospital) (Additional files 1–3).

Women likely to be included in the study were informed about it at their 9th month pregnancy visit, in order to have sufficient time for reflection or at the latest during admission to the delivery room. The information was provided both in writing (posters in the waiting and consultation rooms) and orally by investigators at this visit and in the delivery room if necessary. All participants provided written informed consent. As it was not known in advance whether vacuum extraction would be needed consent forms were signed and collected before knowing if an indication for vacuum extraction would in the end be decided.

In the delivery room, if a vacuum extractor was needed consent was confirmed orally and after verifying eligibility criteria, the woman was electronically randomized by the obstetrician in charge of the delivery and allocated to either the iCup® (‘disposable cup’ group) or to the usual Drapier-Faure® metallic cup (‘metallic cup’ group). Randomization was centralised using a web server and was stratified by center (random block sizes of 6 or 10) to give equal distribution between both groups for each center (ClinInfo SA, Lyon, France).

### Ethics statement

This study was approved by Grenoble regional IRB (Comité de Protection des Personnes Sud Est V) on August 4, 2009 number: 09-CHUG-19.

### Data collection

An electronic-case report form (e-crf) was used to collect demographic and clinical data on the woman and the new-born at baseline and during the whole follow-up period. Maternal and neonatal serious adverse events were also collected.

Investigators accessed the e-crf with individual passwords.

### Primary outcome

The primary criterion was a composite including both the risk of cup dysfunction responsible for a clinical impact (prolongation of delivery and eventually change in mode of delivery) and the most frequent maternal and neonatal harms.

The precise criteria were as follows: the use of other instruments after attempted vacuum extraction, caesarean section after attempted vacuum extraction, three detachments of the cup (considered as failure in French recommendations [4]), episiotomy and perineal tears; and for the new-born: caput succedaneum (swelling of the scalp), cephalohaematoma (an effusion of blood beneath the periosteum of the skull). If one or more of these elements were observed an ‘event’ was considered to have occurred; otherwise ‘no event’ was recorded.

Obstetric data were collected by the obstetrician but were objective well-defined criteria. Pediatric data were recorded by an independent pediatrician.

A composite criterion was chosen because we wanted to include both neonatal and maternal elements in judging the success of the intervention. Nevertheless, we needed to find a balanced compromise between similar severity and the frequency of each element.

The components of this composite endpoint represent the vast majority of clinical risks of instrumental vaginal delivery using a ventouse [3]. Furthermore, the advantage of using a composite endpoint was to be able to immediately assess the potential superiority of the instrument with a sufficient number of events without requiring an excessively large number of patients for the trial.

#### Secondary outcomes

The secondary criteria were maternal and neonatal outcomes that are rare, are minor, or are independent of the type of device used:

- maternal lesions: cervical lesions, postpartum haemorrhage >500 ml and >1000 ml;
- neonatal lesions: minor scalp injuries, Apgar score < 7, pH < 7.20, anaemia (haemoglobin < 14.5 g/dl), jaundice (bilirubin > 150 μmol/l), transfer to NICU.

### Studied medical devices

The iCup® vacuum extraction device is sterile and disposable. It consists of a cup-shaped vacuum chamber measuring 5 cm in diameter and 2.5 cm in height with a base made of medical-grade elastomer, which is relatively flexible, an adjustable traction strap and an ergonomic handle. The inner and outer surfaces of the cup are smooth.

On the dome of the cup there is a furrow specially designed to fit the obstetrician’s finger allowing to locate its position while it is being inserted and during the foetus’s progression. The inside of the cup has an inner roof which is slightly convex, with a protective filter layer of synthetic foam with open pores. The aspiration inside the cup is circumferential to the inner roof in order to avoid any risk of obstruction of the vacuum system. The traction strap, consisting of a metal cable covered by a plastic protective coating with indicators at 5 cm and 10 cm, is located exactly at the centre of the dome of the cup and can be rotated through 360°. It has a total length of 60 cm, and this can be adjusted as required by inserting the end into blocking stops on the handle.

The flexible vacuum tube, which emerges from the dome of the cup, is 1.40 m in length and ends in an adjustable-diameter adaptor designed to fit most of the vacuum pumps on the market. This tube is strengthened by an inner reinforced tube where it emerges from the cup to avoid any risk of obstruction due to kinking during the extraction.

### Statistical analysis

Assuming a frequency of events of at least 35 % with the metallic vacuum cup (standard device) [5], and to detect any relative reduction in frequency with the disposable cup of 30 %; it was necessary to include 330 women per group (i.e. 660 women) for 80 % power and a bilateral alpha risk of 5 % (Calculated using nQuery Advisor 6.01) [9, 10].

Considering that 5 % of women would, in the end, not require vacuum assisted delivery, the number of women to be included was increased to 700, or 350 per group.

The target population was women for whom vacuum extraction was indicated and who were able to undergo this. Randomization was made in an emergency context at installation in the delivery room. It was foreseen in the study protocol that some women would finally not require vacuum assisted delivery and they were to be excluded from the analyses. Indeed, these were not ‘protocol deviations’ (noncompliance or loss to follow-up) but ‘inclusion errors’, which were independent of the treatment or its outcome. This also concerned mothers/babies who in the end were unable to have vacuum assisted extraction (i.e. operator’s decision on the appearance of exclusion criteria to vacuum extraction). The intention to treat analysis was then performed for all other randomized women.

Descriptive statistics included frequencies and percentages for categorical variables with a 95 % confidence interval, and means and standard deviations for continuous variables or medians and the interquartile range for non-Gaussian continuous-level variables.

Univariate analysis was performed when appropriate. Continuous data were compared using a *t*-test if the variable was normally distributed or Mann Whitney test for non-parametric variables. The Chi-square test (Fisher’s exact test if necessary) was used for categorical variables. Statistical significance was considered at p-value ≤0.05.

In this randomized controlled trial, statistical testing for baseline differences between the intervention and control group (Tables 1 and 2) was not done, in line with arguments in the CONSORT statement (http://www.consort-statement.org/checklists/view/32-consort/510-baseline-data; accessed August 2011).

**Table 1:** Clinical characteristics of women at inclusion

	Disposable cup group (*n* = 295)	Metallic cup group (*n* = 283)
Clinical characteristics		
Maternal age (years), mean ± SD	28 ± 5	29 ± 5
Maternal weight (Kg) mean ± SD	73.4 ± 13.1	72.6 ± 13.9
Gestational age (weeks) mean ± SD	40 ± 1	40 ± 1
Nulliparous n (%)	190 (64.4 %)	206 (72.8 %)
Anesthesia n (%)		
Epidural	294 (99.7 %)	278 (98.2 %)
Other (spinal, general anesthesia)	4 (1.4 %)	1 (0.4 %)
Comorbidities n (%)		
Diabetes	14 (4.8 %)	13 (4.6 %)
Hypertension	4 (1.4 %)	5 (1.8 %)
Other morbidities	30 (10.2 %)	23 (8.1 %)

**Table 2:** Obstetrical characteristics of women and fetus at inclusion

	Disposable cup group (*n* = 295)	Metallic cup group (*n* = 283)
Instrumental indication		
Abnormal fetal heart rate pattern	154 (52.2 %)	166 (58.7 %)
No progress of the presentation (arrest of descent in active part of second stage)	220 (74.6 %)	200 (70.7 %)
Maternal indications^b^	23 (7.8 %)	29 (10.3 %)
Station of vertex at cup application	*n* = 286	*n* = 273
Outlet (+3, +4)	57 (19.3 %)	50 (17.7 %)
Low (+2)	229 (77.6 %)	223 (78.8 %)
Position of application of cup	*n* = 295	*n* = 282
LOA (Left Occipital Anterior)	110 (37.3 %)	111 (39.4 %)
ROA (Right Occipital Anterior)	76 (25.8 %)	64 (22.7 %)
OA (Occipital Anterior)	55 (18.64 %)	52 (18.4 %)
ROP (Right Occipital Posterior)	25 (8.5 %)	23 (8.2 %)
Others (OT, LOP, OP)^a^	29 (9.8 %)	32 (11.4 %)
Position at delivery	*n* = 286	*n* = 278
Occipital-anterior	267 (93.4 %)	257 (92.5 %)
Occipital-posterior	19 (6.6 %)	21 (7.6 %)
Duration of labor from 4 cm of dilation to delivery (hour, mean ± SD)	*n* = 292	*n* = 282
	6.7 ± 2.6	6.5 ± 2.9

^a^ *OT* Occipital Transverse, *LOP* Left Occipital Posterior, *OP* Occipital Posterior

^b^Assistance with delivery for women with uterine scarring or a maternal disorder (eclampsia, pre-eclampsia, cardiac pathology, respiratory difficulty, para or tetraplegia, cerebral aneurism, retinopathy etc.)

All statistical analyses were performed using Stata SE version 11.0 software (StataCorp LP, 4905 Lakeway Drive, College Station, Texas 77845-4512, USA).

#### Post-hoc analyses

In order to explore the learning curve for the use of a new medical device we divided the study inclusion period into quartiles (from Q1: first period to Q4: last period of inclusion in the study). While the numbers of experienced, junior and trainee obstetricians were not comparable from one center to another, over the whole study all levels of experience were equally represented.

## Results

**Fig. 1:**
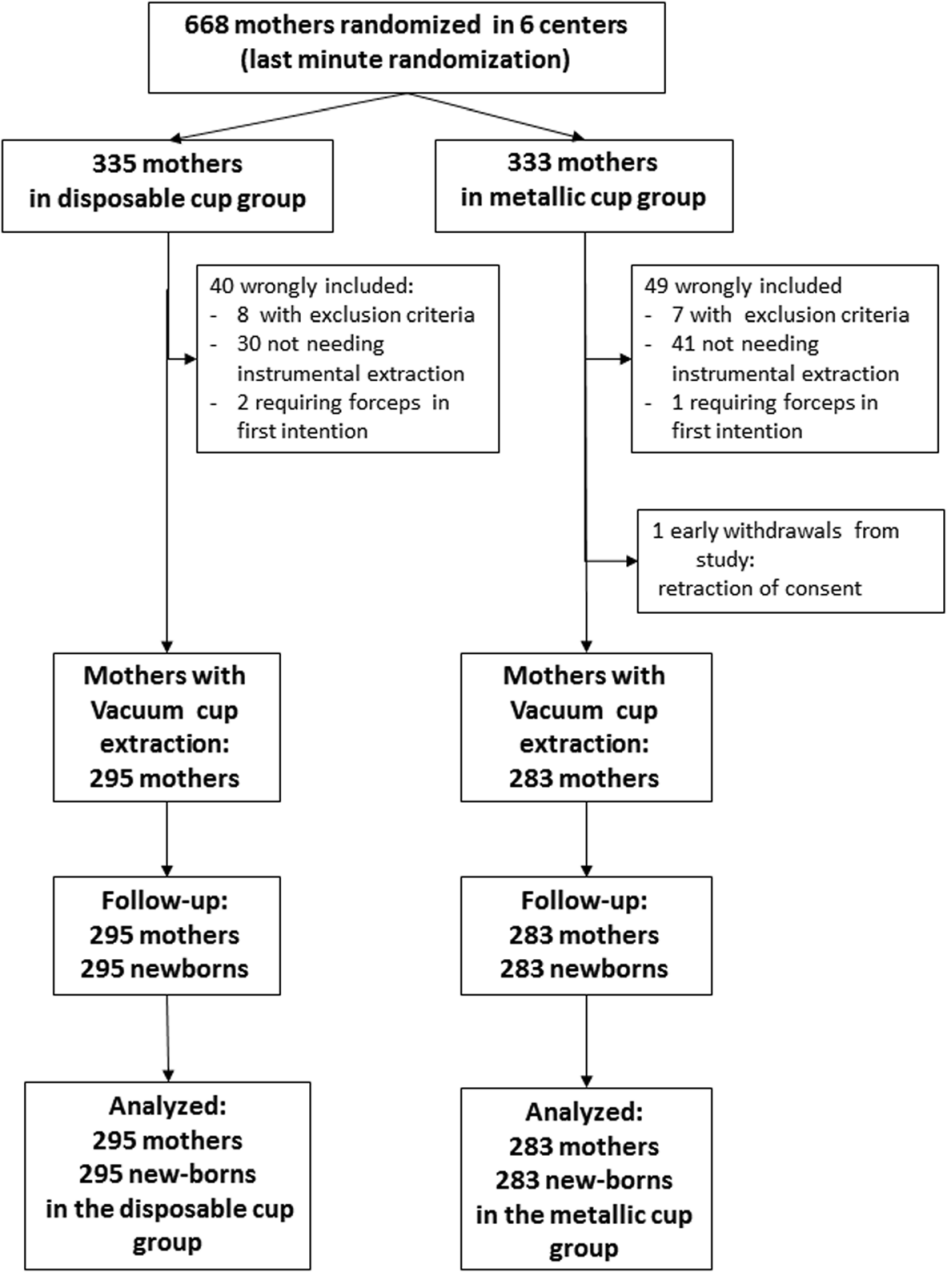
Study flow chart of the new disposable cup

During the study period, 668 women were randomized. Five out of the six centers included between 100 and 150 patients each, whereas the last center included only 42 women. Ninety women who in the end did not meet the inclusion criteria were subsequently excluded, as foreseen in the protocol, and the 578 mothers requiring vacuum extractions and their new-borns were followed and analysed (Fig. 1). These exclusions were similar in both groups and concerned 71 mothers who finally did not require instrumental extraction and 18 mothers unable to have a vacuum extraction (10 women in the ‘disposable cup’ group: two with forceps extraction first and eight with other exclusion criteria; and 9 women in the ‘metallic cup’ group: one with forceps extraction first, seven with other exclusion criteria including one withdrawal of consent).

As presented in Tables 1 and 2, clinical and obstetrical characteristics at inclusion were not different whatever the group, especially the indication for instrumental extraction, position of the vertex at application of cup, and the position of the cup on the scalp at application. Only the proportion of nulliparous women was greater in the ‘metallic cup’ group: 64.4 % versus 72.8 %.

In terms of the primary composite endpoint, the study found no significant difference between the two groups: 76.3 % of events in the ‘disposable cup’ group versus 69.3 % in the ‘metallic cup’ group, *p* = 0.06 (Table 3), the relative risk was 0.91 (95 % CI: 0.82-1.01).

**Table 3:** Primary composite criterion and analysis of its separate elements

	Disposable cup group (*n* = 295)	Metallic cup group (*n* = 283)	*p*-value
Primary composite criterion^a^	225 (76.3 %)	196 (69.3 %)	0.06
Failed delivery with allocated vacuum device^b^	105 (35.6 %)	20 (7.1 %)	0.0001
Forceps whatever the number of detachments	29 (9.8 %)	14 (5.0 %)	0.03
Caesarean section whatever the number of detachments	13 (4.4 %)	3 (1.1 %)	0.01
Other type of vacuum cup whatever the number of detachments	71 (24.1 %)	3 (1.1 %)	<0.01
3 detachments^c^	9 (3.1 %)	1 (0.4 %)	0.02
Neonatal outcomes			
Caput succedaneum	62 (21.0 %)	63 (22.3 %)	NS
Cephalohaematoma	5 (1.7 %)	4 (1.4 %)	NS
Maternal outcomes			
Episiotomy	116 (39.3 %)	108 (38.1 %)	NS
No perineal tears	22 (7.5 %)	7 (2.5 %)	<0.01
First degree perineal tear(s) only	103 (34.9 %)	101 (35.7 %)	NS
Second degree perineal tear(s) only	50 (17.0 %)	58 (20.5 %)	NS
Third degree/fourth degree perineal tears^d^	5 (1.7 %)	14 (5.0 %)	<0.01

^a^At least one event

^b^Nine mothers had at least two attempted modes of delivery (2 F + CS, 1 F+ other cup and 6 CS+ other cup)

^c^Eutocic delivery after 3 detachments

^d^Perineal tears potentially associated with episiotomy (six mothers: 1 in the disposable cup group and 5 in the metallic cup group)

However, an analysis of each element of the primary composite criterion taken separately showed some differences (Table 3). Failed delivery was more frequent in the disposable cup group, mainly due to detachment: 35.6 % (105) vs 7.1 % (20), *p <* 0.0001. Conversely, perineal tears were more frequent in the metallic cup group, especially for third or fourth grade perineal tears: 1.7 % versus 5.0 %, *p =* 0.003.

Table 4 presents the final mode of delivery according to the number of cup detachments and describes precisely the obstetrical practice. In particular it shows that only 10 % concerned ‘three detachments’, in a total of 20 detachments in the disposable cup group and 7 in the metallic cup group. More precisely, when cup detachment was followed by the use of another instrument, about 90 % were employed from the first or second detachment: 92.3 % of cup detachments were followed by forceps, and 87.3 % of cup detachments were followed by a different type of ventouse.

**Table 4:** Final mode of delivery and number of cup detachments

	Number of cup detachments			Total
	1	2	3	
Cup detachment followed by caesarean section (CS)				
Disposable cup group	3	1	0	4
Metallic cup group	0	0	1	1
Cup detachment followed by forceps				
Disposable cup group	10	14	2	26
Metallic cup group	4	4	4	12
Cup detachment followed by another type of cup				
Disposable cup group	30	25	8	63
Metallic cup group	1	1	1	3
Cup detachment followed by other instrument and then CS				
Disposable cup group	3	3	1	7
Metallic cup group	0	1	0	1
Cup detachment followed by vaginal delivery without other instrument^a^				
Disposable cup group	51	29	9	89
Metallic group	38	7	1	46
Total cup detachments^b^				
Disposable cup group (*n* = 295)	97	72	20	189
Metallic cup group (*n* = 283)	43	13	7	63

^a^Not counted in the primary endpoint

^b^Nine mothers had at least two modes of attempted delivery (2 F + CS, 1 F+ other cup and 6 CS+ other cup)

The study found 64.1 % of total cup detachments in the disposable cup group versus 22.3 % in the metallic cup group. If cup detachments followed by another cup or followed by vaginal delivery without other instruments being used were excluded, total cup detachment decreased to 12.5 % in the disposable cup group [189 − (63 + 89)] vs 5.0 % [63 − (3 + 46)].

There were no significant differences concerning the secondary criteria: post-partum haemorrhage, transfer of the new-born to a neonatal intensive care unit (NICU) or serious adverse events (Table 5). Three serious adverse events were reported by the vigilance unit in the disposable cup group (1 postpartum haemorrhage, 1 minor scalp injury and 1 neonatal intracerebral haemorrhage) compared to zero in the metallic cup group, *p =* 0.27.

**Table 5:** Secondary criteria and adverse events

	Disposable cup group (*n* = 295)	Metallic cup group (*n* = 283)	*p*-value
Maternal outcomes			
Postpartum hemorrhage > 500 ml	31 (10.5 %)	25 (8.8 %)	NS
Postpartum hemorrhage > 1000 ml	0 (0.0 %)	0 (0.0 %)	NS
Other maternal adverse event^a^	18 (6.1 %)	19 (6.7)	NS
Neonatal outcomes			
Apgar score <7			
1 minute (294)	38 (13.9 %)	32 (11.3 %)	NS
5 minutes (294)	7 (2.4 %)	6 (2.1 %)	NS
10 minutes (293)	3 (1.0 %)	0 (0.0 %)	NS
Umbilical arterial pH	(*n* = 277)	(*n* = 262)	
pHa < 7.0	5 (1.8 %)	3 (1.1 %)	NS
pHa < 7.20	99 (35.7 %)	89 (34.0 %)	NS
Umbilical venous pH	(*n* = 191)	(*n* = 185)	
pHv < 7.0	0 (0.0 %)	1 (0.5 %)	NS
pHv < 7.20	28 (14.7 %)	20 (10.8 %)	NS
Minor scalp injuries	11 (3.7 %)	14 (5.0 %)	NS
Transfer to Resuscitation unit	25 (8.5 %)	22 (7.8 %)	NS
Anaemia^b^	4 (1.4 %)	2 (0.7 %)	NS
Jaundice^b^	45 (15.3 %)	51 (18.0 %)	NS
other neonatal adverse event^a^	33 (11.2 %)	27 (9.5 %)	NS
Serious adverse event^c^	3 (11.2 %)	0 (0 %)	NS

^a^Prolongation of hospitalization or unit transfer

^b^Event observed at D1 and D3

^c^One maternal and 2 neonatal events reported by the hospital vigilance unit: one postpartum hemorrhage, one minor scalp injury, and one neonatal intracerebral hemorrhage

Although no training with the innovative device had been done before the study, no trends in the learning curve were found during the study. The rates of events per quartile (Q) in the iCup group were respectively: Q1: 73.6 %, Q2: 75.7 %, Q3: 75.3 % and Q4: 80.6 %. In the metallic cup group, event rates were: Q1: 73.5 %, Q2: 73.2 %, Q3: 60.3 % and Q4: 70.4 %.

## Discussion

### Main findings

Concerning the primary composite criterion, the study did not find any significant difference between the two groups, although the p-value was at the limit of significance (6 %). However the analysis of each element of the primary composite criterion taken separately showed significant differences: failure to complete delivery with the allocated ventouse was more frequent in the disposable cup group, mainly due to detachment. Conversely, perineal tears were significantly more frequent in the metallic cup group, especially third or fourth grade perineal tears.

A posteriori, the components of this composite criterion had been well-chosen; apart from these, few other events related to instrumental deliveries were observed (see Table 5).

### Strengths and limitations

The advantage of using a composite endpoint was that we were able to immediately assess the superiority, or not, of the device. Separate risk-benefit assessments would have required two or more trials with all the disadvantages inherent to this: an increase in the number of subjects to be included and especially no result as to the overall effectiveness (or not) of the new cup for several years. This would have been detrimental to the development of the innovative medical device where programmed obsolescence is on average 5 years. In addition, equivalence trials remain controversial, among specialists there is rarely an ‘agreed efficacy loss’ and furthermore there is rarely consensus among specialists. The components of the composite endpoint used here are the criteria most frequently used when assessing the benefit-risk balance of instrumental vaginal deliveries by ventouse [2, 3, 5].

Although the clinical trial was not blinded, it was randomized and it included nearly 600 women in six centres. Thus any patient selection bias by the obstetricians is unlikely (Tables 1 and 2). Moreover, contrary to other trials in the literature, it had a homogeneous control group and is at present the largest multicenter clinical trial on instrumental deliveries [3].

### Comparison with published data

We observed a higher rate of events than expected (76.3 % vs 35 %) explained mainly by the larger number of cup detachments and probably also due to the composite nature of the primary criterion.

Concerning previous reports in the literature on the use of soft vacuum cups: even though we observed more extraction failures, as was expected given the data in the literature on soft versus metallic cups [3, 11–16], we found the same rate of neonatal scalp events despite the soft nature of the new disposable cup material in contact with the neonatal scalp. Nonetheless, we observed fewer perineal tears in the disposable cup group, particularly severe tears, a finding that did not emerge in the Cochrane review of O’Mahony et al.; comparing soft with metallic cups [3]. This might be explained by a better compromise between vacuum cup traction and perineal resistance owing to the ‘mixed’ properties of the new disposable ventouse, in particular its ‘non-lateralised’ aspiration tube.

Concerning rigid disposable versus reusable metallic cups, 4 controlled clinical trials have compare rigid, the Kiwi Omnicup®, with metallic cups (two multicenter and two single center studies) [17–20].

With regard to vacuum cup failures, multicenter studies found similar rates of vacuum cup failure in the metallic group as we did (Attilakos 34 %, Groom 30 % and this study 39.9 %) [17, 18]. However, they had little recourse to another ventouse (Attilakos 3 %, Groom 4 % and this study 24 %) [17, 18].

If we consider the single center studies, the rate of vacuum cup failure was curiously inexistent or very low in groups: Ismail et al. reported no extraction failures in either study group: Kiwi Omnicup® versus Mamstrom metal cup [19]; and Mola and Kuk found only 2 % failure rate in the Omnicup group and 6 % in the Bird metal group [20]. We note that both these studies were small in size (Ismail 164 women, Mola 200 women) [19, 20].

Multicenter studies found no statistical significance in the numbers of perineal tears between the Kiwi Omnicup® device and a metallic cup, whereas in our study there were statistically more severe perineal tears with the metallic cup (5 %) than with the new disposable cup (1.7 %). In the studies the rate of severe perineal tears was greater with the Kiwi Omnicup® device than with the new disposable cup (Attilakos 7 % and Groom 4 %). However, again, both these studies were smaller than our study (Attilakos 200 women, Groom 404 women) [17, 18].

Concerning the occurrence of episiotomy, there was no significant difference between the groups but it has tended to be higher than in our study: Attilakos about 50 %, Groom about 60 % and our study about 30 % whatever the group.

As in the literature, our study did not find significant differences concerning maternal or neonatal serious adverse events.

### Learning curve

The evaluation of an innovative device remains complex particularly if one wants to take into account the learning curve before the study. It increases the cost of the study, is time-consuming and is also difficult to quantify because it varies, in significant ways, from one medical device to another.

There was no ‘run in’ period before our study. Surprisingly, the number of extraction failures did not significantly vary over time (1st quartile - 4th quartile), as might have been expected if there had been a normal learning curve. This result could be explained by a rapid early abandon of the new technology in certain centres where investigators felt more comfortable with more familiar instruments, especially as the control group was homogeneous using a single standard technique that was well tested and had been used routinely by all the investigators over a long period. In fact, the number of extraction failures could be over-estimated all over the study, as the obstetricians quickly abandoned the new device after only one or two detachments (instead of three, as written in the study protocol). The results in Table 4 are in line with this hypothesis.

Few studies have investigated learning curves in obstetrics, and even less have sought to quantify the effect. In the case of vacuum cup devices, a French study aimed at assessing the learning curve of young residents concluded that it was quite short, not more than 6 procedures [21]. More recently, an American simulation trial quantifying subjective levels of traction by the obstetrician using the Kiwi Omnicup® did not find any differences. The authors thus assumed that the learning curve for use of the device was relatively short, as the force applied by junior residents was not different from that of more experienced practitioners [22].

Nevertheless, O’Mahony et al. noted that the previous experience of the obstetrician remains central to the choice of instrument [3].

## Conclusion

This study is to date the largest multicentre randomised controlled trial on instrumental deliveries, with in addition a homogeneous control group. While the new disposable cup had more cup detachments and extraction failures than the standard metallic cups, this innovative disposable device had the advantage of causing fewer perineal injuries.

It would be useful to continue the evaluation of the device with a cost efficacy study and interviews to assess practitioner satisfaction.

## Additional files

Additional file 1:
**CONSORT 2010 checklist of information to include when reporting a randomised trial.** (DOC 217 kb) Additional file 2:
**Experimental Protocole.** (DOCX 55 kb) Additional file 3:
**Protocole Experimental.** (PDF 7021 kb)
