# Supplementary material for: Clinical impact of the disposable ventouse iCup® versus a metallic vacuum cup: a multicenter randomized controlled trial
Source: BMC Pregnancy Childbirth. 2015 Dec 15;15:332. doi: 10.1186/s12884-015-0771-1 (PMC4678725; doi:10.1186/s12884-015-0771-1)
Supplement: Additional file 2: — Experimental Protocole. (DOCX 55 kb) [file 12884_2015_771_MOESM2_ESM.docx]

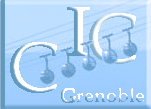

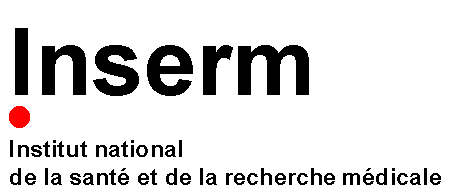

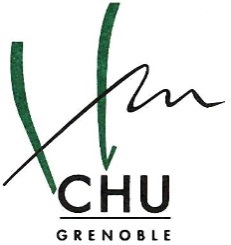


**EXPERIMENTAL PROTOCOLE**

# général Information

| **Title:** **Benefit-risk assessment of a new disposable obstetrical vacuum extractor versus the reference vacuum extractor**: a multicenter randomized, controlled, superiority trial in perinatology  **Short Title:** iCup |
| --- |
| **Methodology:** Multicenter randomized controlled therapeutic trial of superiority: disposable iCup versus sterilizable metal suction cup; randomized in parallel groups, open-label, performed as recommended by the CONSORT group. |
| **Medical Device studied:** disposable obstetrical vacuum extractor iCup |
| **Investigateur coordonnateur autorised à signer le protocol:** Dr Véronique Equy (CHUG), Clinique Universitaire de Gynécologie Obstétrique, CHU de Grenoble (en remplacement du Pr Jean-Patrick Schaal) |
| **Investigateurs :**  - Pr Jean-Patrick SCHAAL, Clinique Universitaire de Néonatologie, CHU de Grenoble  - Dr Véronique EQUY, Clinique Universitaire de Gynécologie Obstétrique, CHU de Grenoble ;  - Pr Didier LEMERY et Dr Françoise VENDITTELLI, Gynécologie-Obstétrique, CHU de Clermont-Ferrand ;  - Dr Victoire CABAUD, Gynécologie-Obstétrique CHG de Chambéry ;  - Pr Didier RIETHMULLER, Gynécologie-Obstétrique, CHU de Besançon ;  - Pr Michel DREYFUS, Gynécologie-Obstétrique, CHU de Caen ;  - Pr Bruno LANGER, Gynécologie-Obstétrique, CHU de Strasbourg. |
| **Methodologists :**  - Dr Sandra DAVID-TCHOUDA, Cellule de soutien et d’évaluation médico-économique aux innovations, CHU de Grenoble ;  - Pr Jean-Luc BOSSON, Centre d’Investigation Clinique de Grenoble. |
| **Industriel :**  M. François GUERIN : Société Collin |
| **Sponsor:** CHU de Grenoble  **Sponsor’s representative authorized to sign the protocol:** Mme Hélène SABBAH-GUILLAUME  Direction de la Recherche Clinique et de l’Innovation, CHU de Grenoble, 38043 Grenoble Cedex 09  Tel : 04 76 76 59 57 Fax : 04 76 76 52 21 |
| **Study Coordonnating Center:**  Centre d’Investigation Clinique – Inserm 003**,** CHU de Grenoble, 38043 Grenoble Cedex 09  Tel : 04 76 76 92 60 Fax : 04 76 76 92 62 |

# protocol SUMMARY

| **PROTOCOL – SUMMARY** |
| --- |
| **Study Centers:**  Pôle Couple - Enfant, CHU de Grenoble (Grenoble University Hospital);  Service de Gynécologie - Obstétrique, CHU de Clermont-Ferrand ;  Service de Gynécologie - Obstétrique, CHG de Chambéry ;  Service de Gynécologie - Obstétrique, CHU de Besançon ;  Service de Gynécologie - Obstétrique, CHU de Caen ;  Service de Gynécologie - Obstétrique, CHU de Strasbourg. |
| **Co-ordinnating Investigator authorized to sign the protocol :** Dr Véronique Equy (CHUG), cCinique Universitaire de Gynécologie Obstétrique, CHU de Grenoble (will replace Pr Jean-Patrick Schaal) |
| **Investigators :**  - Pr Jean-Patrick SCHAAL, Gynécologie-Obstétrique, CHU de Grenoble  - Dr Véronique EQUY, Gynécologie-Obstétrique, CHU de Grenoble;  - Dr Françoise VENDITTELLI, Gynécologie-Obstétrique, CHU de Clermont-Ferrand ;  - Dr Victoire CABAUD, Gynécologie-Obstétrique CHG de Chambéry ;  - Pr Didier RIETHMULLER, Gynécologie-Obstétrique, CHU de Besançon ;  - Pr Michel DREYFUS, Gynécologie-Obstétrique, CHU de Caen ;  - Pr Bruno LANGER, Gynécologie-Obstétrique, CHU de Strasbourg. |
| **Methodologists:**  - Dr Sandra DAVID-TCHOUDA, Innovations medico-economic evaluation unit, Grenoble University Hospital  - Pr Jean-Luc Bosson, Grenoble Clinical Research Center. |
| **Industriel:**  M. François GUERIN : Collin |
| **Sponsor:** Grenoble University Hospital |
| **Methodology:** Multicenter randomized controlled therapeutic trial of superiority: disposable iCup versus sterilizable metal suction cup; randomized in parallel groups, open-label, performed as recommended by the CONSORT group.  **Calculation of study size:**  Assuming a frequency of harmful events to mother and/or newborn of 35% with the reusable metal suction cup (routinely used in French obstetrics units), and to demonstrate a relative reduction in this frequency with the disposable iCUP vacuum extractor cup of 30%; it is necessary to include 330 patients per group, i.e. a total of 660 patients (for a power of 80% and an alpha risk of 5%).  Considering a potential rate of refusal to participate in the study of 5%, the number of patients to be included is 700, or 350 per group.  Due to the innovative nature of the medical device, the first description of the elements of the composite endpoint will be performed at 200 patients to verify the assumptions regarding the frequency of events and to adjust the calculation of the number of subjects needed, if necessary. |
| **Main objective:** Assess the risk-benefit balance of the new obstetrical vacuum extractor iCUP, in terms of morbidity of mothers and infants and functioning of the medical device, compared to the reusable metal suction cup (standard treatment).  **Main outcome:** this is a composite endpoint combining the risk of failure of the medical device (detachment or failure of extraction) and expected benefits in terms of morbidity of mother and child:   - Detachment of the medical device, - Failure of extraction: use of forceps or caesarean - Occurrence of one of the following fetal complications: cephalohematoma, caput succedaneum, - Occurrence of one of the following maternal complications: episiotomy, perineal tears (with involvement of the anal sphincter or not.)   The components of the composite endpoint are clinically objective criteria.  In addition, there will always be a double validation: neonatal data will be validated by the obstetrician and the neonatologist, a doctor independent of the instrumental delivery; and monitoring of maternal obstetric complications by the obstetrician and a midwife.  Finally, quality control of 100% of the data will be conducted for the main outcome from the medical records |
| **Secondary Objective 1:** To assess the effectiveness of the new iCup vacuum extractor in terms of rare or minor morbidities of the mother and child compared to the reusable metal cup (standard treatment).  1 / the following complications occurring in the mother not necessarily linked to the medical device: cervical tears, postpartum haemorrhage;  2 / the following complications occurring in the fetus not necessarily linked to the medical device: poor neonatal outcome (Apgar score <7, pH <7.20), anemia, jaundice, or transfer to neonatal intensive care unit.  3 / the following minor complications occurring to the fetus: of the scalp  **Secondary Objective 2:** The following elements of the composite endpoint: failures up to the failure of extraction, episiotomy and caput succedaneum, will each be evaluated separately.  **Secondary Objective 3:** to perform a cost-effectiveness analysis of the new iCup vacuum extractor ICUP compared to the reusable metal cup (standard treatment) from the point of view of the hospital. |
| **Main inclusion criteria:**   - Age between 18 and 45 years - Woman delivering a single living child after 37 weeks GA, with cephalicpresentation, for which there is an indication for instrumental extraction using a vacuum extractor.   Person covered by the French social security scheme or equivalent |
| **Medical device studied:** single use disposable iCup vacuum extractor |
| **Reference Medical device:** reusable metal vacuum extractor |
| **Total number of subjects:** 700 randomized subjects |
| **Total study duration:** 3 years |
| **Study duration for the subject:** 4 days on average, up to 28 days (neonatal period) |
| **Safety Criteria:** Clinical Reports of all adverse events. An independent surveillance committee will meet periodically throughout the duration of the study to monitor security related to the medical device studied. It will monitor adverse events. The committee will decide on any possible termination of the study |
