# Supplementary material for: Clinical impact of the disposable ventouse iCup® versus a metallic vacuum cup: a multicenter randomized controlled trial
Source: BMC Pregnancy Childbirth. 2015 Dec 15;15:332. doi: 10.1186/s12884-015-0771-1 (PMC4678725; doi:10.1186/s12884-015-0771-1)
Supplement: Additional file 3: — Protocole Experimental. (PDF 7021 kb) [file 12884_2015_771_MOESM3_ESM.pdf]

Inserm
Institut national
de la santé et de la recherche médicale

CHU GRENOBLE

PROTOCOLE EXPERIMENTAL

1. INFORMATIONS GENERALES

**Titre : Evaluation du rapport bénéfice/risque d'une nouvelle ventouse obstétricale à usage unique versus la ventouse obstétricale de référence**<br>Essai de supériorité randomisée, contrôlée, multicentrique en périnatalogie<br>**Titre court :** iCup
**Méthodologie :** Essai thérapeutique de supériorité, randomisé en groupes parallèles, contrôlé (ventouse obstétricale à usage unique iCup versus ventouse métallique à usage multiple), multicentrique, en ouvert**,** réalisé suivant les recommandations du CONSORT group.
**Dispositif médical à l'étude :** ventouse obstétricale à usage unique iCup
**Investigateur coordonnateur autorisé à signer le protocole:** Dr Véronique Equy (référent du CHUG), Clinique Universitaire de Gynécologie Obstétrique, CHU de Grenoble (en remplacement du Pr Jean-Patrick Schaal)
**Investigateurs :**<br>- Pr Thierry DEBILLON, Clinique Universitaire de Néonatologie, CHU de Grenoble<br>- Dr Véronique EQUY, Clinique Universitaire de Gynécologie Obstétrique, CHU de Grenoble ;<br>- Pr Didier LEMERY et Dr Françoise VENDITTELLI, Gynécologie-Obstétrique, CHU de Clermont-Ferrand ;<br>- Dr Victoire CABAUD, Gynécologie-Obstétrique CHG de Chambéry ;<br>- Pr Didier RIETHMULLER, Gynécologie-Obstétrique, CHU de Besançon ;<br>- Pr Michel DREYFUS, Gynécologie-Obstétrique, CHU de Caen ;<br>- Pr Bruno LANGER, Gynécologie-Obstétrique, CHU de Strasbourg.
**Méthodologistes :**<br>- Dr Sandra DAVID TCHOUDA, Cellule de soutien et d'évaluation médico-économique aux innovations, CHU de Grenoble ;<br>- Pr Jean-Luc BOSSON, Centre d'Investigation Clinique de Grenoble.
**Industriel :**<br>M. François GUERIN : Société Collin
**Promoteur :** CHU de Grenoble<br>**Représentant du promoteur autorisé à signer le protocole** : Mme Hélène SABBAH-GUILLAUME<br>Direction de la Recherche Clinique et de l'Innovation, CHU de Grenoble, 38043 Grenoble Cedex 09<br>Tel : 04 76 76 59 57 Fax : 04 76 76 52 21
**Centre coordonnateur de l'étude :**<br>Centre d'Investigation Clinique - Inserm003**,** CHU de Grenoble, 38043 Grenoble Cedex 09<br>Tel : 04 76 76 92 60 Fax : 04 76 76 92 62

2. RESUME DU PROTOCOLE

PROTOCOLE – RESUME
**Centres d'étude :**<br>Pôle Couple - Enfant, CHU de Grenoble ;<br>Service de Gynécologie - Obstétrique, CHU de Clermont-Ferrand ;<br>Service de Gynécologie - Obstétrique, CHG de Chambéry ;<br>Service de Gynécologie - Obstétrique, CHU de Besançon ;<br>Service de Gynécologie - Obstétrique, CHU de Caen ;<br>Service de Gynécologie - Obstétrique, CHU de Strasbourg.
**Investigateur coordonnateur autorisé à signer le protocole :** Dr Véronique Equy (référent du CHUG), clinique Universitaire de Gynécologie Obstétrique, CHU de Grenoble (en remplacement du Pr Jean-Patrick Schaal)
**Investigateurs :**<br>- Dr Véronique EQUY, Gynécologie-Obstétrique, CHU de Grenoble ;<br>- Pr Jean-Patrick SCHAAL, Gynécologie-Obstétrique, CHU de Grenoble ;<br>- Dr Françoise VENDITTELLI, Gynécologie-Obstétrique, CHU de Clermont-Ferrand ;<br>- Dr Victoire CABAUD, Gynécologie-Obstétrique CHG de Chambéry ;<br>- Pr Didier RIETHMULLER, Gynécologie-Obstétrique, CHU de Besançon ;<br>- Pr Michel DREYFUS, Gynécologie-Obstétrique, CHU de Caen ;<br>- Pr Bruno LANGER, Gynécologie-Obstétrique, CHU de Strasbourg.
**Méthodologistes :**<br>- Dr Sandra DAVID TCHOUDA, Cellule de soutien et d'évaluation médico-économique aux innovations, CHU de Grenoble ;<br>- Pr Jean-Luc Bosson, Centre d'Investigation Clinique de Grenoble.
**Industriel :**<br>M. François GUERIN : Société Collin
**Promoteur :** CHU de Grenoble
**Méthodologie :** Essai thérapeutique de supériorité, randomisé en groupes parallèles, contrôlé (ventouse iCup versus ventouse métallique à usage multiple), multicentrique, en ouvert, réalisé suivant les recommandations du CONSORT group.<br><br>En faisant l'hypothèse d'une fréquence des évènements morbides du couple mère-enfant à 35 % avec la ventouse métallique à usage multiple (traitement de référence), et pour mettre en évidence une réduction relative de fréquence avec la ventouse iCup de 30 % ; il est nécessaire d'inclure 330 patientes par groupe soit 660 patientes (pour une puissance de 80 % et un risque alpha à 5 %).<br>En considérant un taux de refus de participer à l'étude de 5 % le nombre de patientes à inclure est de 700, soit 350 par groupe.<br>En raison du caractère innovant du DM, une première description des éléments du critère composite sera réalisée à 200 patientes, afin de vérifier les hypothèses de départ concernant la fréquence des évènements et réajuster le calcul du nombre de sujets si nécessaire.

**Objectif principal :** Évaluer la balance bénéfice-risque de la nouvelle ventouse obstétricale iCup, en termes de morbidité du couple mère-enfant et fonctionnement du dispositif médical, comparée à la ventouse métallique à usage multiple (traitement de référence).<br>**Critère de jugement principal :** il s'agit d'un critère composite alliant le risque d'échec du dispositif médical (lâchage ou échec de l'extraction) et les bénéfices attendus sur la morbidité du couple mère-enfant. :<br>- lâchage du dispositif médical,<br>- échec d'extraction : utilisation de forceps ou césarienne,<br>- survenue des complications fœtales suivantes : céphalhématome, bosse séro-sanguine,<br>- survenue des complications maternelles suivantes : épisiotomie, déchirures périnéales (avec atteinte du sphincter anal ou non)<br>Les éléments du critère composite sont cliniquement objectifs.<br>En outre, il existera systématiquement une double validation : les données néonatales seront validées par l'obstétricien et le néonatologue, médecin indépendant de l'extraction instrumentale ; le suivi des complications maternelles obstétricales, par l'obstétricien et la sage-femme.<br>Enfin, un contrôle qualité de 100% des données sera réalisé pour le critère de jugement principal à partir du dossier médical.
**Objectif secondaire 1** : Évaluer l'efficacité de la nouvelle ventouse obstétricale iCup sur la morbidité rare ou mineure du couple mère-enfant comparée à la ventouse métallique à usage multiple (traitement de référence).<br>**Critères de jugement de l'objectif secondaire 1 :**<br>1/ les complications suivantes survenant chez la mère n'étant pas forcément lié au DM : déchirures cervicales, hémorragies de la délivrance ;<br>2/ les complications suivantes survenant chez le fœtus n'étant pas forcément lié au DM : mauvais état néonatal (score d'Apgar < 7, pH < 7,20), anémie, ictère, et transfert néonatal.<br>3/ les complications mineures suivantes survenant chez le fœtus : excoriations du cuir chevelu.<br><br>**Objectif secondaire 2** : les éléments du critère composite suivants (défaillances du DM allant jusqu'à l'échec d'extraction, épisiotomie et bosse séro-sanguine) seront évalués chacun séparément.<br><br>**Objectif secondaire 3** : réaliser une analyse cout-efficacité de la nouvelle ventouse obstétricale iCup comparée à la ventouse métallique à usage multiple (traitement de référence) du point de vue de l'hôpital.
**Critères d'inclusion principaux** :<br>- Age compris entre 18 et 45 ans<br>- Patiente accouchant d'un enfant vivant unique après 37 SA, en présentation céphalique, pour laquelle il existe une indication d'extraction instrumentale par ventouse obstétricale.<br>Personne affilié à la sécurité sociale ou bénéficiaire d'un tel régime
**Dispositif médical à l'étude :** ventouse obstétricale à usage unique iCup
**Dispositif médical de référence :** ventouse métallique à usage multiple
**Nombre total de sujets :** 700 sujets randomisés
**Durée totale de l'étude :** 3 ans
**Durée de l'étude par sujet :** 4 jours en moyenne, 28 jours maximum (période néonatale)
**Critères de sécurité :** Recueil clinique des évènements indésirables<br>Un comité de surveillance indépendant se réunira périodiquement durant toute la durée de l'étude pour contrôler la sécurité liée au traitement à l'étude. Il monitorera les évènements indésirables. Ce comité pourra statuer sur un éventuel arrêt de l'étude.

SOMMAIRE

1. INFORMATIONS GENERALES 1
2. RESUME DU PROTOCOLE 2
3. JUSTIFICATION DE L'ÉTUDE 6
3.1. Presentation generale du probleme 6
3.2. Justification de l'etude compte tenu des connaissances actuelles. 7
3.3. Hypotheses de recherche 8
4. OBJECTIF(S) DE L'ÉTUDE 8
4.1. Objectif principal : 8
4.2. Objectifs secondaires : 10
5. DISPOSITIFS MEDICAUX COMPAREES 11
5.1. Dispositif medical evalue 11
5.2. Dispositif medical de reference 12
5.3. Circuit des dispositifs avec la Pharmacie Hospitaliere 12
6. CARACTERISTIQUES DES SUJETS 12
6.1. Modalites de recrutement des sujets 12
6.2. Criteres d'inclusion 12
6.3. Criteres de non-inclusion 13
6.4. Traitements associes autorises ou interdits 13
7. ORGANISATION GENERALE DE L'ETUDE 13
7.1. Type d'etude 13
7.2. Deroulement pratique de la randomisation 13
7.3. Deroulement pratique de l'etude 13
7.4. Procedures d'investigation menees et differences par rapport a la prise en charge habituelle 14
7.5. Lieux de l'etude 15
8. MODE DE RECUEIL ET GESTION DES DONNEES 15
8.1. Recueil des donnees 15
8.2. Gestion des donnees 16
8.3. Monitorage des donnees / gestion de la Qualite des donnees 16
9. VARIABLES MESURÉES ET MÉTHODES DE MESURE 16
9.1. Parametres cliniques 16
9.2. Parametres biologiques 18
10. ANALYSE STATISTIQUE DES PARAMÈTRES MESURÉS 18
10.1. Calcul du nombre de sujets 18
10.2. Strategie d'analyse des donnees 19
10.2.1. Description de la population étudiée 19
10.2.2. Analyse du critère de jugement principal 19
10.2.3. Analyse des critères de jugement secondaires cliniques 19
10.2.4. Analyse des critères de jugement secondaires médico-économiques 20
10.3. Responsable des analyses 20
10.4. Lieu d'analyse des donnees et logiciels utilises 20
11. CALENDRIER PREVISIONNEL DE L'ÉTUDE 20
12. EVALUATION DE LA SECURITE 20
12.1. Definition d'un evenement indesirable (EI) 20
12.2. Definition de l'evenement indesirable grave (EIG) 21
12.3. Definition d'un EI inattendu 21
12.4. EI dus aux ventouses 21
12.5. Modalites de detection et de documentation d'un EIG 22
12.6. Personnes a prevenir en cas de survenue d'un EI 22
12.7. Procedure en cas de defaillance d'un dispositif medical 22
13. ARRÊT PRÉMATURÉ DE L'ÉTUDE 22

13.1. CRITERES D'ARRET DE L'ETUDE POUR UN SUJET QUI Y PARTICIPE 22
13.2. ARRET DE L'ETUDE PAR LE PROMOTEUR 22
13.3. ARRET DE L'ETUDE PAR L'INVESTIGATEUR 22
14. ASPECTS MATÉRIELS ET LÉGAUX 23
14.1. INFORMATION DU PATIENT - CONSENTEMENT ECLAIRE ECRIT 23
14.2. SECRET PROFESSIONNEL, CONFIDENTIALITE 23
14.3. CONTRAT D'ASSURANCE 23
14.4. FINANCEMENT DE L'ETUDE 23
14.5. COMITE DE PROTECTION DES PERSONNES DANS LA RECHERCHE BIOMEDICALE 23
14.6. AMENDEMENTS AU PROTOCOLE 24
14.7. ANONYMAT DES SUJETS PARTICIPANT A L'ETUDE 24
14.8. ASSURANCE QUALITE 24
14.9. AUTORISATION DE LIEU DE LA RECHERCHE 24
14.10. FICHIER NATIONAL DES RECHERCHES BIOMEDICALES ET PERIODE D'EXCLUSION 24
14.11. PUBLICATIONS 24
14.12. ARCHIVAGE 24
15. DONNÉES TECHNIQUES SUR LE MATÉRIEL TESTÉ 25
15.1. DESCRIPTION 25
15.2. INDICATIONS 25
15.3. CONTRE-INDICATIONS 26
15.4. MODE OPERATOIRE 26
16. RÉFÉRENCES BIBLIOGRAPHIQUES 29
17. ANNEXES 31
17.1. ANNEXE A: FORMULAIRE D'INFORMATION A LA PATIENTE 31
17.2. ANNEXE B: CONSENTEMENT DE PARTICIPATION 35
17.3. ANNEXE C : BUDGET 36
17.4. ANNEXE D : NOTICE ICUP 38
17.5. ANNEXE E : DECLARATION DE CONFORMITE DES VENTOUSES OBSTETRICALES ICUP 44
17.6. ANNEXE F : ATTESTATION CE ICUP 45
17.7. ANNEXE G : TABLEAU D'IDENTIFICATION DES INVESTIGATEURS REFERENTS 46
17.8. ANNEXE H : CV DES INVESTIGATEURS REFERENTS 47
17.9. ANNEXE I : LISTE DES INVESTIGATEURS 53
17.10. ANNEXE J : DELEGATIONS DE TACHES 55
17.11. ANNEXE K : ETUDE DE L'ICUP EN LABORATOIRE 59
17.12. ANNEXE L : FICHE DE DECLARATION D'EVENEMENT INDESIRABLE 67

3. JUSTIFICATION DE L'ÉTUDE

3.1. Présentation générale du problème

La ventouse obstétricale encore appelée « vacuum extractor » est un dispositif médical qui, comme le forceps et les spatules, permet d'aider la femme à enfanter par les voies naturelles [1].

Jusque dans les années 1950, le seul instrument disponible pour les extractions fœtales était le forceps. L'apparition de la ventouse obstétricale a donné la possibilité de choisir entre deux instruments.

L'évolution des taux d'utilisation de ces deux instruments dans le monde, souvent en faveur de la ventouse, a donné une idée des préférences des obstétriciens 1-22.

Depuis la diffusion de la ventouse obstétricale dans les pays européens nordiques, son usage est pratiquement exclusif en Suède et au Danemark [2]. La ventouse est également populaire en Afrique et en Asie [3]. En Angleterre, Amérique du Nord et du Sud et en Australie, si la pénétration de la ventouse obstétricale a été plus lente, l'augmentation croissante de l'utilisation de la ventouse a aussi été largement décrit [3-5]. Plusieurs publications nord-américaines ont montré, l'utilisation majoritaire de la ventouse obstétricale [6, 7]. Depuis 1992, aux États-Unis, le taux d'utilisation de la ventouse est devenu majoritaire [8] [9-12]. En 2004, le taux d'utilisation (calculé sur 4,1 millions de naissances) de la ventouse aux États-Unis était de 4,1 % des naissances et celui des forceps de 1,1 % [10]. En 2005, ce taux d'utilisation (calculé sur 1,2 millions de naissances) était de 3,6 % des naissances et celui des forceps de 0,9 % [13]. Cette utilisation majoritaire de la ventouse obstétricale a également été constatée en Angleterre [14-16] et au Canada [17]. En Australie, en 1997, l'utilisation de la ventouse par rapport au forceps est passée de 1/6 en 1990 à 1/1 [18, 19] et sur une publication de 2005, sur 267 793 patientes, le taux de forceps était de 3,5 % et le taux de ventouse de 7,2 % [20].

En France, le taux d'utilisation de la ventouse obstétricale représentait, en 1993, 13,37 % des extractions instrumentales. Son usage était majoritaire en Alsace, Franche-Comté, Basse-Normandie, Auvergne et Languedoc-Roussillon [21]. Dans la banque de données Audipog, sur 14 937 accouchements en 2004-2005, on a décrit un taux de forceps à 5,1 %, de ventouses à 3 % et de spatules à 3 % (www.audipog.net). Actuellement, les extractions instrumentales sont utilisées dans environ 10 % à 15 % des 860 000 accouchements annuels et l'usage de la ventouse augmente d'année en année. Enfin, plusieurs autres facteurs de bonnes pratiques, ou simplement « société », expliquent l'augmentation croissante de l'utilisation de la ventouse, notamment la mise au ban de la méthode d'« expression abdominale » par l'HAS en 2007 recommandant en particulier l'utilisation de la ventouse [22], ou le ressenti négatif des forceps par les parents, ou les recommandation de l'OMS privilégiant la diminution des césariennes dont le nombre est toujours supérieur à celui recommandé (http://www.who.int/reproductive-health/mcpc_fr/interventions/i_51_62_cesarienne.html).

Dès lors, en plus des ventouses métalliques stérilisables associées à une pompe électrique, différents types de ventouses se sont multipliés, notamment la ventouse Kiwi à usage unique ou les ventouses dites « soft ». Or, les études les évaluant n'ont pas montré leur supériorité par rapport à la ventouse classique métallique stérilisable.

Les complications avec la ventouse Kiwi étaient comparables à celles observées avec les ventouses métalliques mais avec un taux d'échec supérieur aux ventouses métalliques [23-25]. Pour les ventouses « soft » les complications fœtales étaient moins importantes mais le taux d'échec étaient très supérieur à celui des ventouses métalliques [26].

En conséquence, il est légitime d'évaluer la ventouse iCup, « mélange » innovant des ventouses métalliques et des ventouses « soft » qui devrait réunir les avantages des deux ; et de faire une comparaison entre la ventouse iCup et les ventouses métalliques stérilisables qui sont les plus performantes du marché à ce jour.

3.2. Justification de l'étude compte tenu des connaissances actuelles.

La ventouse iCup stérile à usage unique a été conçue selon un cahier des charges prenant en compte les différents avantages et inconvénients des différentes ventouses actuellement utilisée sur le marché.

Elle fonctionne sur la même base de caractéristique que la Minicup, ventouse métallique stérilisable utilisée le plus souvent. Minicup est une ventouse métallique stérilisable à usage multiple (forme en cupule, diamètre 5 cm, hauteur environ 1,5 cm).

La « cupule » de dispositif iCup (figure 1) est construite en trois parties en plastique : un dôme collé à une base (jupe) et un toit interne fixé au fond du dôme. S'attachent au centre du dôme un lac de traction et sa poignée. S'attache sur l'orifice d'entrée du vide du toit dans la cupule un tube d'aspiration qui débouche dans la partie arrière de celui-ci. La base en contact avec le scalp du crâne du fœtus de façon étanche utilise un matériau souple moins traumatisant que celui des cupules métalliques ou plastiques actuelles [26].

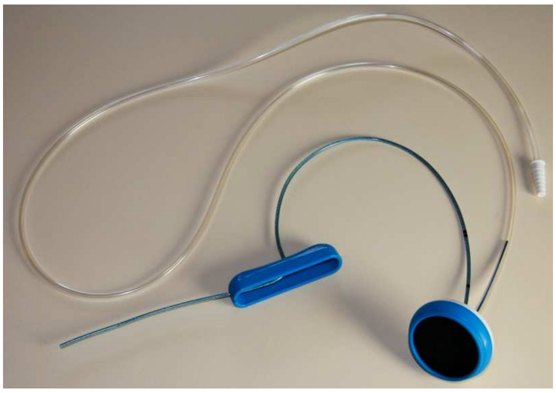

Figure 1 : ventouse iCup®

Le taux d'échec reconnu des ventouses existantes est lié à leur construction qui n'est plus conforme aux besoins actuels de l'opérateur accoucheur [26]. Les besoins sont triples : 1/ éviter que la ventouse dérape et se décolle (lâchage) à cause d'une traction mal dirigée, ou d'une construction non adhérente et donc non étanche pour maintenir le vide selon les paramètres physiques requis, 2/ éviter que la ventouse soit traumatisante à cause de matériau trop rigide au contact du crâne du fœtus ou de forme intérieure protubérante, 3/ éviter que la ventouse ne soit bouchée par un caillot de sang.

Les améliorations de la ventouse iCup sont nombreuses et prennent en compte les besoins de l'obstétricien cités ci-dessus : 1/ la base souple de la ventouse iCup utilise le SANTOPRENE un polyuréthane permet un contact plus « doux » avec le scalp du fœtus, 2/ le toit fixé a 2 avantages : l'intérieur du dôme a une forme concave lisse vers l'extérieur non traumatisante pour la mère, il permet une répartition et une circulation du vide uniforme dans la cupule afin d'éviter que l'orifice d'accès du vide ne soit bouché par un caillot de sang ; 3/ le dôme comporte une encoche de positionnement de forme adaptée au doigt de l'opérateur lui permettant d'orienter la cupule, l'effort mécanique attaché au dôme en son centre permet à l'opérateur d'exercer une traction

contrôlée centrale et perpendiculaire au dôme et donc au scalp du fœtus, seule méthode pour éviter un échec. En outre, le lac de traction attaché au dôme en son centre comporte une gaine plastique sur laquelle sont imprimées deux marques de positionnement à 5 cm et 10 cm du sommet du dôme pour permettre à l'opérateur de savoir comment progresse l'extraction. Il est muni d'une poignée ajustable pour s'adapter aux besoins ergonomiques de l'opérateur. Cette poignée de construction particulière permet de se fixer au câble à la longueur désirée avec une double sécurité (taquet bloquant) sans gêner l'action de l'opérateur. Enfin, le tube d'aspiration au sommet du dôme est perpendiculaire au dôme, il est doublé par un tube intérieur renforcé sur 10 cm pour éviter une pliure qui l'obstruerait.

Des expérimentations portant sur le fonctionnement de l'iCup ont été effectuées 1/ en laboratoire pour contrôler le produit et 2/ sur mannequin pour tester la technique qui ne diffère en rien de la technique habituellement utilisée avec les ventouses métalliques utilisées (Annexe E). Une étude pilote observationnelle sur l'iCup, débutée dans le service de gynécologie-obstétrique du CHU de Grenoble en novembre 2008, après obtention du marquage CE (N° CE 0456), devrait nous donner des premiers résultats de fonctionnement en 2009.

3.3. Hypothèses de recherche

Nous formulons l'hypothèse que la ventouse iCup, dont les caractéristiques physiques font augurer d'une innocuité supérieure (diminution des complications survenant chez la mère et chez l'enfant), est plus efficace que la ventouse métallique stérilisable, pour un taux de fonctionnement (lâchage et échec d'extraction) équivalent.

L'objectif principal de cette étude est donc d'évaluer le rapport bénéfice-risque de la nouvelle ventouse obstétricale « iCup » - i.e. description du taux d'échec des ventouses : lâchage ou échec d'extraction, associée à la description de la santé du couple mère-enfants (complications materno-fœtales) ; en le comparant à la ventouse de référence actuelle la plus performante (la ventouse métallique stérilisable associée à une pompe électrique).

4. OBJECTIF(S) DE L'ÉTUDE

4.1. Objectif principal :

Objectif principal : Évaluer la balance bénéfice-risque de la nouvelle ventouse obstétricale iCup, en termes de morbidité du couple mère-enfant et de fonctionnement du dispositif médical (lâchage ou échec d'extraction), comparée à la ventouse métallique à usage multiple associé à une pompe électrique (instrument de référence).

Critère de jugement principal: il s'agit d'un critère composite alliant le risque d'échec (lâchage du dispositif médical ou échec de l'extraction) et les bénéfices sur la morbidité du couple mère-enfant :

- lâchage du dispositif médical (3 maximum),
- échec d'extraction : utilisation de forceps ou césarienne,
- survenue des complications suivantes du scalp : céphalhématome, bosse séro-sanguine,
- survenue des complications maternelles suivantes : épisiotomie, déchirures périnéales (avec atteinte du sphincter anal ou non)

Si un élément au moins du critère composite survient pendant l'accouchement, le critère composite sera noté « échec » sinon il sera noté « succès ».

Justification du critère composite :

Les éléments constituant le critère composite ci-dessus représentent la grande majorité des critères de la balance bénéfices-risques d'un accouchement voie basse instrumental avec ventouse [27, 28]. L'avantage de ce critère composite est de pouvoir évaluer d'emblée la supériorité de l'instrument. Séparer la balance bénéfice-risque contraignait à réaliser 2 essais : tout d'abord un essai d'équivalence évaluant le fonctionnement des 2 ventouses (risque d'échec d'extraction principalement) ; puis un essai de supériorité portant sur la morbidité du couple mère-enfant (bénéfices attendus princeps). La mise en œuvre des 2 essais nécessitait plus de sujets, entrainait des coûts plus élevés et une absence de résultats portant sur l'efficacité globale de la ventouse avant plusieurs années. En outre, l'essai d'équivalence est de plus en plus controversé, face à une « perte consentie d'efficacité » (i.e. la borne inférieure d'équivalence adoptée par le(s) clinicien(s)) qui est rarement consensuelle dans la spécialité [29]).

Enfin, les éléments constituant le critère composite sont de gravité et/ou conséquences comparables ce qui nous permet de les réunir de façon raisonnable.

Validation du critère de jugement principal :

Les éléments du critère composite sont cliniquement objectifs.

En outre, il existera systématiquement une double validation : les données néonatales seront validées par l'obstétricien et le néonatologue, médecin indépendant de l'extraction instrumentale ; le suivi des complications maternelles obstétricales, par l'obstétricien et la sage-femme.

Enfin, un contrôle qualité de 100% des données sera réalisé pour le critère de jugement principal à partir du dossier médical.

4.2. Objectifs secondaires :

Objectif secondaire 1 : Évaluer **l'efficacité** de la nouvelle ventouse obstétricale iCup sur la morbidité rare ou mineure du couple mère-enfant comparée à la ventouse métallique à usage multiple (traitement de référence).

Critères de jugement de l'objectif secondaire 1 :

1/ les complications suivantes survenant chez la mère n'étant pas forcément lié au DM : déchirures cervicales, hémorragies de la délivrance ;

2/ les complications suivantes survenant chez le fœtus n'étant pas forcément lié au DM : mauvais état néonatal (score d'Apgar < 7, pH < 7,20), anémie, ictère, et transfert néonatal ;

3/ les complications mineures suivantes survenant chez le fœtus : excoriations du cuir chevelu.

Objectif secondaire 2 : les éléments du critère composite suivants (défaillances du DM allant jusqu'à l'échec d'extraction, épisiotomie et bosse séro-sanguine) seront évalués chacun séparément.

Objectif secondaire 3 : réaliser une analyse cout-efficacité de la nouvelle ventouse obstétricale iCup comparée à la ventouse métallique à usage multiple (traitement de référence) du point de vue de l'hôpital.

Critères de jugement de l'objectif secondaire 3 :

1/ les couts seront estimés à partir des critères suivants :

- quantité de ventouses utilisées par accouchement,
- cout unitaire de la ventouse Icup (prix du marché),
- couts unitaires des ventouses stérilisables métalliques : cout du DM (prix du marché), cout de la stérilisation, nombre moyen d'utilisation par ventouse.

2/ les conséquences (mesure d'effet) seront estimées à parti des critères d'efficacité suivants :

- critère de jugement principal : critère composite alliant le risque d'échec (lâchage du dispositif médical ou échec de l'extraction) et les bénéfices sur la morbidité du couple mère-enfant,
- et critère de jugement secondaire 1 : morbidité rare ou mineure du couple mère-enfant.

5. DISPOSITIFS MEDICAUX COMPAREES

5.1. Dispositif médical évalué

La « cupule » de dispositif iCup est construite en trois parties en plastique (**figure 2**) : un dôme collé à une base (jupe) et un toit interne fixé au fond du dôme. S'attachent au centre du dôme un lac de traction et sa poignée. S'attache sur l'orifice d'entrée du vide du toit dans la cupule un tube d'aspiration qui débouche dans la partie arrière de celui-ci. La base en contact avec le scalp du crâne du fœtus de façon étanche utilise un matériau souple moins traumatisant que celui des cupules métalliques ou plastiques.

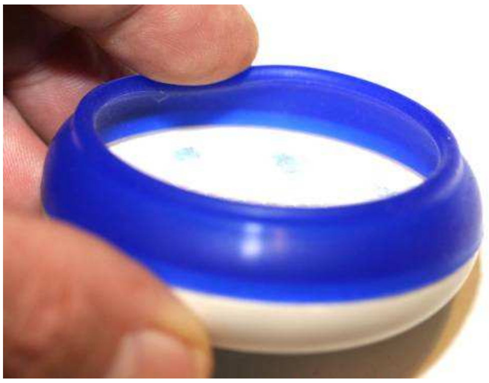

Figure 2 : ventouse iCup®

Les améliorations de la ventouse iCup sont nombreuses et concernent les 3 éléments de la ventouse :

1/ la base de la ventouse iCup qui utilise le SANTOPRENE un polyuréthane permettant un contact plus « doux » avec le scalp du fœtus,

2/ le toit fixé qui a 3 avantages : l'intérieur du dôme a une forme concave lisse vers l'extérieur non traumatisante pour la mère, il permet une répartition et une circulation du vide uniforme dans la cupule afin d'éviter que l'orifice d'accès du vide ne soit bouché par un caillot de sang de plus une interface poreuse commune à de nombreux modèles de ventouses a été conservé pour accroître la sécurité fœtale.

3/ le dôme comporte une encoche de positionnement de forme adaptée au doigt de l'opérateur lui permettant d'orienter la cupule, l'effort mécanique attaché au dôme en son centre permet à l'opérateur d'exercer une traction contrôlée centrale et perpendiculaire au dôme et donc au scalp du fœtus, seule méthode pour éviter un échec.

En outre, le lac de traction attaché au dôme en son centre comporte une gaine plastique sur laquelle sont imprimées deux marques de positionnement à 5 cm et 10 cm du sommet du dôme pour permettre à l'opérateur de savoir comment progresse l'extraction. Il est muni d'une poignée ajustable pour s'adapter aux besoins ergonomiques de l'opérateur. Cette poignée de construction particulière permet de se fixer au câble à la longueur désirée avec une double sécurité (taquet bloquant) sans gêner l'action de l'opérateur

Enfin, le tube d'aspiration au sommet du dôme est perpendiculaire au dôme pour éviter une pliure qui l'obstruerait

Les données médicales portant sur les indications, contre-indications, et risques du DM, ainsi que les données techniques portant sur la procédure d'utilisation et les mesures de précaution, sont rédigées de manière exhaustive au chapitre 15 et en annexe B.

5.2. Dispositif médical de référence

La ventouse de référence dans l'étude sera la ventouse métallique et stérilisable associée à une pompe électrique type ventouse de Collin (cf. annexe B : attestation de conformité).

5.3. Circuit des dispositifs avec la Pharmacie Hospitalière

La pharmacie hospitalière de chaque centre recevra les ventouses à usage unique iCup, ainsi que les ventouses métalliques stérilisables. Tous les dispositifs seront alors dispensés globalement aux services de gynécologie obstétrique concernés. Aucun retour ne sera fait à la pharmacie.
Le circuit pour la stérilisation des ventouses à usage multiple sera le même qu'utilisé habituellement hors essai.

6. CARACTERISTIQUES DES SUJETS

6.1. Modalités de recrutement des sujets

Les femmes susceptibles d'être incluses dans l'étude seront informées à la consultation du 9ème mois : l'information sera écrite (affiches placardées dans les salles d'attente et dans les salles de consultation) et orale par le médecin investigateur, il répondra de fait aux questions de la patiente.
Afin d'avoir un délai de réflexion suffisant, celles qui souhaitent participer à l'étude, en cas de nécessité de recours à une ventouse obstétricale lors du travail, signeront le consentement de participation en salle de naissance. Le médecin investigateur pourra compléter l'information donnée au 9ème mois si la patiente le souhaite.

Au moment de l'accouchement, si l'obstétricien juge utile l'emploi d'une ventouse, la patiente sera alors randomisée dans l'un des deux groupes : ventouse à usage unique versus ventouse conventionnelle. Sur 1 000 femmes ayant signé le consentement, on s'attend à ce que 10 % soient éligibles et incluses car ayant besoin d'une ventouse, les autres seront éligibles mais non randomisées.
Le recueil des données se fera dans les services de gynécologie obstétrique et néonatalogie des 6 centres impliqués.

6.2. Critères d'inclusion

Seront proposés pour l'étude les sujets répondant à chacun des critères suivants :

- Age compris entre 18 et 45 ans,
- Patiente accouchant d'un enfant vivant unique après 37 SA, en présentation céphalique, pour laquelle il existe une indication d'extraction instrumentale par ventouse obstétricale,
- Personne affilié à la sécurité sociale ou bénéficiaire d'un tel régime.

6.3. Critères de non-inclusion

Ne pourront pas être inclus les sujets répondant à au moins un des critères suivants :

- refus de consentement,
- patiente accouchant avant 37 SA.,
- grossesse multiple,
- présentation non céphalique,
- Personne privée de liberté par décision judiciaire ou administrative, personne faisant l'objet d'une mesure de protection légale.

6.4. Traitements associés autorisés ou interdits

Tous les traitements prescrits usuellement lors d'un accouchement et dans les jours qui suivent seront autorisés.

Aucun traitement habituellement utilisé ne sera interdit du fait de la participation à cette étude.

7. ORGANISATION GENERALE DE L'ETUDE

7.1. Type d'étude

Essai de supériorité, randomisé en groupes parallèles, contrôlé, multicentrique, en ouvert ; réalisé suivant les recommandations du CONSORT group (www.consort-statement.org).

7.2. Déroulement pratique de la randomisation

La randomisation sera centralisée et à disposition soit sur un serveur internet (accès par code sécurisé 24H/24), soit à l'aide d'enveloppes scellées numérotées. Le mode de randomisation (internet, enveloppes) sera testé en salle de naissance, avant le début de l'étude (été 2009), afin de déterminer le mode qui a une faisabilité optimale dans ces conditions d'urgence. La société Clininfo® gèrera l'élaboration et la mise à disposition de la randomisation sur cahier observation électronique.

Les patientes seront alors réparties de manière aléatoire dans l'un des deux groupes. L'allocation se fera par bloc de tailles aléatoires 6 ou 10, avec une stratification par centre.

La randomisation ne sera faite qu'après information donnée à la patiente et signature du consentement.

7.3. Déroulement pratique de l'étude

L'étude se déroulera sur 3 ans.

- La durée planifiée des inclusions sera de 2 ans ½
- La durée planifiée du suivi des patientes sera de 4 jours en moyenne par patiente, 28 jours maximum.

Les femmes susceptibles d'être incluses dans l'étude seront informées à la consultation du 9ème mois : l'information sera écrite (affiches placardées dans les salles d'attente et dans les salles de consultation) et orale par le médecin investigateur, il répondra de fait aux questions de la patiente.

Afin d'avoir un délai de réflexion suffisant, celles qui souhaitent participer à l'étude, en cas de nécessité de recours à une ventouse obstétricale lors du travail, signeront le consentement de participation en salle de naissance. Le médecin investigateur pourra compléter l'information donnée au 9ème mois si la patiente le souhaite.

Au moment de l'accouchement, si l'obstétricien juge utile l'emploi d'une ventouse, la patiente sera alors randomisée dans l'un des deux groupes : ventouse à usage unique versus ventouse conventionnelle.

Tableau synoptique de la chronologie des visites et des examens

	Période de l'essai (J0 à J28 maximum)		
Visites cliniques	En salle d'accouchement	Au passage en Unité mère enfant / dans les 24H suivant l'accouchement	A la sortie de la maternité
Délai par rapport à la randomisation	J0	J1	J « 4 -- 28 »
Information patiente	?		
Critères d'inclusion /exclusion	?		
Formulaire de consentement signé	?		
Données maternelles	?	?	?
Anamnèse		?	
Traitements associés		?	
Caractéristiques du travail	?		
Indications et caractéristiques de l'extraction instrumentale	?		
Taux d'Hémoglobine maternelle		??(J1 ou J2)	
Données néonatales	?	?	?
pH au cordon (A et V)	?		

7.4. Procédures d'investigation menées et différences par rapport à la prise en charge habituelle

Dans cette étude, quel que soit le type de ventouse utilisée (iCup ou ventouse de référence), la prise en charge des patientes ne différera pas de la prise en charge habituelle.

Seul le type de ventouse utilisé sera différent.

7.5. Lieux de l'étude

Clinique de gynécologie Obstétrique, CHU de Grenoble

Clinique de néonatalogie, CHU de Grenoble

Service de Gynécologie-Obstétrique, CHU de Clermont-Ferrand

Service de Gynécologie-Obstétrique, CHG de Chambéry

Service de Gynécologie-Obstétrique, CHU de Besançon

Service de Gynécologie-Obstétrique, CHU de Caen

Service de Gynécologie-Obstétrique, CHU de Strasbourg

8. MODE DE RECUEIL ET GESTION DES DONNEES

8.1. Recueil des données

Le recueil des données se fera par l'intermédiaire d'un cahier d'observation électronique ce qui permet un contrôle qualité des données en temps réel (contrôle à la saisie plus contrôle immédiat par un attaché de recherche clinique avec demandes de correction rapides).

L'accès au cahier d'observation est sécurisé par un identifiant et un mot de passe. Ce cahier d'observation électronique est géré par la Société Clininfo® à Lyon et mis à disposition auprès du centre de coordination avec un logiciel de data management comme support (détection erreurs, formulaires manquants, traçabilité corrections …). Le cahier d'observation électronique permet également un suivi très précis des inclusions (relance des centres adaptée) et donc un pilotage de fin d'étude ainsi qu'une amélioration de la qualité des données.

La faisabilité de ce type d'approche a été démontré en réanimation par le succès de l'étude DOLOREA à laquelle ont participé une quarantaine de services d'anesthésie réanimation au niveau national ayant inclus (étude épidémiologique simple) plus de 1 400 patients en 12 mois avec un taux de formulaires en erreur inférieur à 3 %, ce qui témoigne d'une grande qualité des données [30].

Le cahier d'observation électronique sera rempli par l'investigateur.

Le fichier informatique (données médicales saisies sur le cahier d'observation électronique) sera anonymisé : il ne comportera ni le nom, ni le prénom, ni la date de naissance (simplement l'âge et le sexe), ni d'indication sur la domiciliation des patients. Ce fichier informatique (données médicales) bénéficie de la procédure simplifiée MR001 contractée entre le CHU et la CNIL (n° 117 48 18).

L'analyse statistique ne sera effectuée qu'après vérification de la saisie et de la cohérence des données (procédure gel de base).

8.2. Gestion des données

Toutes les données de l'interrogatoire et des examens cliniques seront présentées dans un dossier spécifique au protocole, consultable par le promoteur, qui constituera les données sources.

Par son accord de participation, l'investigateur s'engage au strict respect du protocole expérimental, des "Bonnes Pratiques Cliniques" et de la législation en vigueur. Il se porte garant de l'authenticité des données recueillies dans le cadre de l'étude et accepte les dispositions légales autorisant le promoteur de l'étude à mettre en place un contrôle de la qualité.

Les données seront archivées par le coordonnateur de l'étude.

8.3. Monitorage des données / gestion de la Qualité des données

Un monitorage des données sera effectué sur les données par un Attaché de Recherche Clinique de la DRCI du CHU de Grenoble ou du centre de coordination (CIC de Grenoble). Il portera sur 15% des cahiers d'observation tirés au sort dans chaque centre recruteur.

L'ensemble des consentements sera vérifié, ainsi que 100 % des données pour les critères d'inclusion/non inclusion, le critère de jugement principal et les évènements indésirables.

Un compte rendu de monitorage sera rédigé par l'ARC et conservé dans le dossier d'étude (promoteur + investigateur).

Un code d'accès au CRF électronique sera prévu pour l'ARC promoteur.

9. <u>VARIABLES MESURÉES ET MÉTHODES DE MESURE</u>

9.1. Paramètres cliniques

<u>A. le jour de l'accouchement, à J0 :</u>

Seront recueillies les critères suivants :

1) Caractéristiques maternelles démographiques et cliniques : âge maternel, parité, gestité, terme, type de grossesse, commune de résidence.

2) Caractéristiques concernant le mode de travail : mode d'anesthésie, durée du travail.

3) Indication et caractéristiques de l'extraction instrumentale :

- indication (anomalie du rythme cardiaque fœtal, non progression de la présentation, raisons maternelles),
- type et hauteur de la présentation,
- durée de l'extraction en minutes,
- lâchages du dispositif médical (critère de jugement principal)

- échec d'extraction : utilisation de forceps, césarienne en urgence (critère de jugement principal).
- l'utilisation finale ou non de la ventouse (changement de pratique médicale en urgence avant utilisation de la ventouse)

4) Données néonatales :

- poids de naissance, périmètre crânien
- complications fréquentes du scalp : céphalhématome ou bosse séro-sanguine (critères de jugement principal), excoriation cutanée du cuir chevelu (critère de jugement secondaire)
- complications rares survenant chez le fœtus : mauvais état néonatal (score d'Apgar < 7, pH < 7,20), anémie, ictère, et transfert néonatal (critères de jugement secondaires)
- autres complications rares.

5) Etat maternel :

- douleur périnéale lors de l'extraction (EVA),
- épisiotomie : réalisation d'une incision périnéale chez la mère (critère de jugement principal).
- état périnéal maternel : périnée intact, déchirure périnéale (1er degré, 2e degré, épisiotomie, 3e degré ou périnée complet, 4e degré ou périnée complet compliqué) (critères de jugement principal),
- hémorragie de la délivrance : poche de recueil de sang en ml (critères de jugement secondaires),
- autre complication du post-partum (infection, maladie thromboembolique, …).

B. de J1 à J28 maximum :

Un examen médical de l'enfant et de la mère sera effectué au 1er jour et à la sortie de la maternité, c'est-à-dire à J1, puis entre J4 : sortie habituelle du couple mère-enfant et J28 : date de point de l'étude. L'état périnéal, la présence ou non de fièvre et l'évaluation de la douleur maternelle à l'aide d'une échelle visuelle analogique (EVA) seront renseignés chaque jour.

Seront donc recueillies les critères suivants :

4) Données néonatales :

- apparition ou évolution des complications fréquentes du scalp : céphalhématome, bosse séro-sanguine (critères de jugement principal), excoriation cutanée du cuir chevelu (critère de jugement secondaire)
- apparition ou évolution des complications rares chez le fœtus : mauvais état néonatal (score d'Apgar < 7, pH < 7,20), anémie, ictère, et transfert néonatal (critères de jugement secondaires)
- apparition ou évolution d'autres complications rares.

5) Etat maternel :

- évolution de la douleur périnéale (EVA),

- évolution de l'état périnéal maternel : périnée intact, déchirure périnéale (1er degré, 2e degré, épisiotomie, 3e degré ou périnée complet, 4e degré ou périnée complet compliqué) (critère de jugement principal),
- apparition ou évolution d'autre complication du post partum (infection, maladie thromboembolique…).

9.2. Paramètres biologiques

Les paramètres suivants sont habituellement recueillis et ne nécessitent pas de prise en charge particulière :

- Le pH au sang du cordon à la naissance veineux et artériel sera mesuré ainsi que le base exces, à l'aide de l'appareil de pHmètre, en salle de naissance
- La numération sanguine : l'hémoglobine est mesurée par une méthode spectrophotométrique après lyse des hématies.

Les tubes de NFS adressés directement aux laboratoires du CHU seront étiquetés avec les étiquettes habituelles du CHU.

10. ANALYSE STATISTIQUE DES PARAMÈTRES MESURÉS

10.1. Calcul du nombre de sujets

En faisant l'hypothèse d'une fréquence des évènements morbides du couple mère-enfant à 35 % avec la ventouse métallique à usage multiple (traitement de référence) [27, 28], et pour mettre en évidence une réduction relative de fréquence avec la ventouse iCup de 30 % ; il est nécessaire d'inclure 330 patientes par groupe soit 660 patientes (pour une puissance de 80 % et un risque alpha à 5 %) (Calcul réalisé par le Dr Sandra David Tchouda sur le logiciel nQuery Advisor 6.01 [31] [32]).

En considérant un taux de refus de participer à l'étude de 5 % le nombre de patientes à inclure est de 700, soit 350 par groupe.

En raison du caractère innovant du DM, une première description des éléments du critère composite sera réalisée à 200 patientes, afin de vérifier les hypothèses de départ concernant la fréquence des évènements et réajuster si nécessaire le calcul du nombre de sujets.

Les accouchements voie basse instrumentaux représentent 10 % au minimum des accouchements [33]. Le recrutement des 700 sujets sur deux ans 1/2 est réaliste avec les 6 centres inclus dans cette étude :

- Le CHU de Grenoble réalise 2 000 accts / an : recrutement potentiel de 200 femmes ;
- Le CHU de Clermont-Ferrand : 2 000 accts / an : recrutement potentiel : 200 femmes ;
- Le CHG de Chambéry : 2 000 accts / an : recrutement potentiel : 200 femmes ;
- Le CHU de Besançon : 2 000 accts / an : recrutement potentiel : 200 femmes ;
- Le CHU de Strasbourg : 2 000 accts / an : recrutement potentiel : 200 femmes ;

- Le CHU de Caen : 3 000 accts / an : recrutement potentiel : 300 femmes ;

Le recrutement potentiel minimum global des 6 centres est de 1 300 femmes sur un an ou 1 950 femmes sur 1 an ½.

10.2. Stratégie d'analyse des données

L'analyse en intention de traiter sera la règle.

10.2.1. Description de la population étudiée

Un diagramme de flux des sujets sera réalisé afin de décrire les effectifs des patientes dans chaque groupe et à chaque étape de l'essai, dès l'inclusion dans l'étude. Ce diagramme permettra de décrire des écarts au protocole potentiels et de décrire la fréquence d'extraction instrumentale sur la population éligible.

10.2.2. Analyse du critère de jugement principal

Les résultats seront décrits en nombre absolu et en fréquence.

La comparaison de fréquence du critère composite suivant la ventouse utilisée sera évaluée à l'aide d'un test du chi-2. Les résultats seront présentés sous la forme d'un risque relatif (RR) encadré de son intervalle de confiance à 95%, ainsi que la réduction relative du risque (RRR) et le nombre de sujet à traiter (NNT).

Chaque élément du critère composite sera décrit séparément dans chaque groupe. Les éléments suivants (défaillances du DM allant jusqu'à l'échec d'extraction, épisiotomie et bosse séro-sanguine) seront testés chacun séparément.

En faisant l'hypothèse d'une fréquence des évènements morbides du couple mère-enfant à 35 % avec la ventouse métallique à usage multiple (traitement de référence) [27, 28], nous souhaitons mettre en évidence une réduction relative de fréquence avec la ventouse iCup de 30 %.

10.2.3. Analyse des critères de jugement secondaires cliniques

La comparaison de moyenne des critères secondaires quantitatifs **(score d'Apgar moyen, PH fœtal au cordon, base exces fœtal, EVA douleur de la mère)** suivant la ventouse utilisée sera obtenue à l'aide d'un test de Student (ou test non paramétriques si l'évènement est rare). Les moyennes seront décrites dans chaque groupe assorties de leur écart-type.

La comparaison de fréquence suivant la ventouse utilisée des autres critères secondaires, qualitatifs, sera obtenue à l'aide d'un test du chi-2 (ou test de Fisher si l'évènement est rare). Les taux seront décrits dans chaque groupe assortis de leur intervalle de confiance à 95 %. Les résultats seront décrits en nombre absolu et en fréquence.

10.2.4. Analyse des critères de jugement secondaires médico-économiques

Après avoir décrit les coûts moyens des 2 DM et l'efficacité chez les patients, on calculera le rapport coût-efficacité différentiel suivant : (coût DM innovant – coût référence) / (efficacité DM innovant – efficacité référence)
Une analyse de sensibilité nous permettra de tester la robustesse de nos estimations.

10.3. Responsable des analyses

Les rédacteurs du plan d'analyse statistique sont :

- Dr Sandra DAVID TCHOUDA, Cellule de soutien et d'évaluation médico-économique aux innovations, CHU de Grenoble ;
- Pr Jean-Luc Bosson, Centre d'Investigation Clinique de Grenoble

Le méthodologiste chargé de réaliser l'analyse est le Dr Sandra DAVID TCHOUDA, Cellule de soutien et d'évaluation médico-économique aux innovations, CHU de Grenoble.

10.4. Lieu d'analyse des données et logiciels utilisés

Les analyses statistiques seront réalisées par le Centre d'Investigation Clinique de Grenoble. L'analyse statistique sera réalisée à l'aide du logiciel Logiciel STATA® version 10 ou supérieure (Stata Corporation 4905 Lakeway Drive College Station, TX 77845 USA)

11. CALENDRIER PREVISIONNEL DE L'ÉTUDE

- Durée de l'étude par couple mère-enfant : 4 jours en moyenne sauf complications. En cas de complications maternelles ou fœtales, le couple mère-enfant sera suivi jusqu'à 28 jours au maximum (fin de la période néonatale).
- Durée totale de l'étude : 3 ans
- Durée des inclusions : 2 ans ½ maximum
- Date prévue pour le début des inclusions : Septembre-octobre 2009
- Date prévue pour la fin de l'étude : Septembre-octobre 2012

12. EVALUATION DE LA SECURITE

12.1. Définition d'un évènement indésirable (EI)

Toute manifestation médicale nocive survenant chez une personne qui se prête à une recherche biomédicale que cette manifestation soit liée ou non à la recherche ou au dispositif médical sur lequel porte cette recherche.

Un EI peut être tout signe défavorable et inattendu (incluant des résultats anormaux de laboratoires), tout symptôme ou pathologie temporaire associé à l'utilisation du dispositif médical, avec ou sans relation avec le dispositif médical.

12.2. Définition de l'évènement indésirable grave (EIG)

Un EIG est un évènement indésirable, avec ou sans relation avec le DM, ayant une des conséquences suivantes : décès, mise en jeu du pronostic vital, hospitalisation ou prolongation d'hospitalisation, invalidité ou incapacité, cliniquement significative, temporaire ou permanente, anomalie ou malformation congénitale.

12.3. Définition d'un EI inattendu

Tout EI du dispositif médical dont la nature, la sévérité ou l'évolution ne concorde pas avec les informations figurant dans le résumé des caractéristiques du dispositif médical lorsqu'il est autorisé et dans la brochure investigateur lorsqu'il n'est pas autorisé.

12.4. EI dus aux ventouses

Les EI dus à la ventouse iCup sont identiques à ceux de la ventouse de référence et sont les suivants :

1/ <u>Atteintes maternelles</u>

La ventouse obstétricale s'applique sur le crâne fœtal et n'augmente pas ses dimensions. Elle évite les problèmes inhérents à une anesthésie générale. La majorité des études ont attribué à la ventouse moins de conséquences délétères pour les parties molles de la femme que le forceps. Les lésions rencontrées sont celles entraînées par un mauvais contrôle du dégagement ou par une mauvaise vérification de la mise en place du dispositif.

- Atteintes vaginales, vulvaires et périnéales
- Atteintes rares : périnées complets ou compliqués, lésions cervicales, hémorragies
- Atteintes évitables : atteintes de la muqueuse vaginale dont un repli était resté pincé entre la cupule et la présentation, atteintes cervicales (si la ventouse a été posée avant la dilatation cervicale complète) qui relevait du même mécanisme.
- Atteintes exceptionnelles : détachement annulaire du col, fistules vésico-vaginale ou vésico-utérine
- Atteintes à distance : incontinence anale et/ou urinaire

2/ <u>Atteintes néonatales</u>

- Atteintes du scalp fœtal : bosse séro-sanguine, excoriation du cuir chevelu, décollement cutané localisé, céphalhématome ou hématome sous-périosté, hématome sous cutané du cuir chevelu
- Autres complications : hémorragies cérébro-méningées, hémorragies rétiniennes, fractures du crâne, ictère, infections, lésions neurologiques

12.5. Modalités de détection et de documentation d'un EIG

Tous les EIG seront notifiés respectivement par l'investigateur au promoteur et par le promoteur à l'autorité compétente (Afssaps). Les EIG inattendus seront déclarés également au Comité de protection des Personnes. Tous les EIG seront consignés et déclarés au CRPV (selon la procédure de référence (PRO /002).

12.6. Personnes à prévenir en cas de survenue d'un EI

Dr Céline Villier - Centre Régional de Pharmacovigilance
CVillier@chu-grenoble.fr
Pharmacovigilance@chu-grenoble.fr
Fax : 04 76 76 56 55 **Tel** : 04 76 76 51 45

12.7. Procédure en cas de défaillance d'un dispositif médical

En cas de défaillance d'une ventouse, l'investigateur changera de matériel par remplacement identique, sans nouvelle randomisation.

Le DM défectueux sera conservé et stocké pour évaluer la défaillance, si nécessaire, suivant la procédure de la pharmacovigilance du CHU de Grenoble (centre promoteur). En ce qui concerne la ventouse iCup, toute défaillance sera notifiée au CRPV et le DM transmis au fabricant pour expertise.

La défaillance sera consignée dans le bordereau « DM défectueux ».

13. ARRET PREMATURE DE L'ETUDE

13.1. Critères d'arrêt de l'étude pour un sujet qui y participe

- Sujet qui retire son consentement de participation à l'étude.

Le retrait du consentement sera notifié dans le « bordereau d'arrêt prématuré de l'étude ».

- EIG selon la définition (Cf. Ch. 12) ; la relation cause-effet sera appréciée par l'investigateur puis le comité de surveillance. L'EIG sera reporté sur le « bordereau des événements indésirables ».

13.2. Arrêt de l'étude par le promoteur

Le promoteur peut arrêter l'étude à tout moment, pour les raisons suivantes : incapacité de l'investigateur à inclure les patients selon le calendrier prévu, absence de consentement signé, violations majeures au protocole, données incomplètes ou erronées et en cas d'EIG.

13.3. Arrêt de l'étude par l'investigateur

En cas d'EI jugé sévère par l'investigateur et pouvant mettre en jeu la santé des patients, l'investigateur peut arrêter l'étude en accord avec le promoteur.

14. ASPECTS MATÉRIELS ET LÉGAUX

14.1. Information du patient - Consentement éclairé écrit

Conformément aux Bonnes Pratiques Cliniques et aux dispositions légales en vigueur, tout sujet présélectionné sera préalablement informé par l'investigateur des objectifs de l'étude, de sa méthodologie, de sa durée, de ses contraintes et des risques prévisibles (EI). Il sera notamment précisé aux patientes qu'elles sont entièrement libres de refuser de participer à l'étude ou de retirer leur consentement à tout moment sans encourir aucune responsabilité ni aucun préjudice de ce fait. Un document résumant les renseignements donnés par l'investigateur leur sera remis (cf. annexe A).

L'information des femmes potentiellement éligibles sera double : 1/ lors de leur consultation du 9ème mois : l'information sera écrite (affiches placardées dans les salles d'attente et dans les salles de consultation) et orale par le médecin investigateur ou la sage-femme ; puis 2/ à l'entrée en salle de naissance.

Après un délai de réflexion et après s'être assuré de la bonne compréhension des informations fournies, l'investigateur sollicitera de la patiente son consentement écrit pour participer à l'étude. Si elle accepte, la patiente signera le formulaire de consentement (cf. annexe A) préalablement à la réalisation de l'étude.

14.2. Secret professionnel, confidentialité

L'investigateur est tenu au respect du secret professionnel. Les données recueillies, y compris les résultats des analyses, seront rendues anonymes par tout moyen approprié. Le promoteur et ses représentants sont soumis aux mêmes obligations de secret professionnel que l'investigateur.

Le présent document et ses annexes sont remis à l'investigateur à titre confidentiel et ne doivent être remis ou communiqués qu'aux personnes nommément impliquées dans l'essai avec l'accord ou à la demande du coordinateur.

14.3. Contrat d'assurance

N° de contrat : 126.959

Société Hospitalière d'Assurances Mutuelles / SHAM

18 rue Edouard Rochet

69372 LYON cedex

14.4. Financement de l'étude

Cette étude bénéficie d'un financement dans le cadre de l'appel d'offre PHRC interrégional 2009.

14.5. Comité de Protection des Personnes dans la Recherche Biomédicale

C.P.P Sud-Est V, Rez-de-Chaussée Haut, C.H.U. de Grenoble, 38043 Grenoble Cedex 09

14.6. Amendements au protocole

La prolongation éventuelle de l'essai fera l'objet d'un amendement soumis pour avis au C.P.P, et à l'autorité compétente.

14.7. Anonymat des sujets participant à l'étude

Les bordereaux des cahiers d'observation ne porteront que les initiales de la patiente (deux premières lettres du nom et la première lettre du prénom) et un numéro d'anonymat. Seules ces données d'identification seront informatisées. Le fichier informatisé utilisé pour la saisie et le traitement des données bénéficie de la procédure simplifiée MR001 contractée entre le CHU et la CNIL (n° 117 48 18).

14.8. Assurance qualité

La partie clinique de l'étude sera réalisée en accord avec les Bonnes Pratiques Cliniques en partenariat avec le CIC.

14.9. Autorisation de lieu de la recherche

Selon l'article L1121-13 du CSP, aucune autorisation de lieu n'est requise pour cette étude ; celle-ci ne nécessite pas des actes autres que ceux pratiqués usuellement dans le service hospitalier où se déroulera l'étude. De plus les personnes se prêtant à l'étude ne présentent pas de condition clinique distincte de celle pour laquelle le service est compétent.

14.10. Fichier national des recherches biomédicales et période d'exclusion.

Les femmes se prêtant à cette étude pourront participer simultanément à d'autres recherches biomédicales.

A l'issu de leur participation à cette étude il n'est pas prévu de période au cours de laquelle elles ne pourront pas participer à une autre recherche biomédicale.

14.11. Publications

Toutes les données recueillies au cours de cette étude sont la propriété du promoteur de l'étude et ne peuvent être communiquées en aucun cas à une tierce personne sans l'accord écrit de l'investigateur coordonnateur et du promoteur.

Toute publication ou communication (orale ou écrite) sera décidée d'un commun accord entre les investigateurs et respectera les recommandations internationales: "Uniforms Requirements for Manuscripts Submitted to Biomedical Journals" (http://www.cma.ca/publications/mwc/uniform.htm)

14.12. Archivage

L'ensemble des dossiers de l'étude sera archivé pour une durée de 15 ans par l'investigateur coordonnateur, sous la responsabilité du promoteur.

Les documents source, les cahiers d'observation, les originaux des formulaires de consentement, le protocole signé devront être conservés par l'investigateur coordonnateur pendant la durée minimale de 15 ans à compter de la fin de l'étude.

L'investigateur coordonnateur organise, au nom du promoteur de l'étude, le stockage dans des locaux appropriés les documents suivants :

- Protocole avec annexes, amendements.
- Cahiers d'observation (originaux) avec documents annexes.
- Document de suivi de l'étude clinique.
- Toutes les pièces administratives et correspondances liées à l'étude.
- Rapport d'étude.

15. DONNÉES TECHNIQUES SUR LE MATÉRIEL TESTÉ

15.1. Description

La ventouse obstétricale iCup est un dispositif d'extraction instrumentale du fœtus (cf. notice en annexe C). Elle est stérile à usage unique, prête à l'emploi dans les conditions d'utilisation et pour les indications recommandées.

La ventouse iCup comprend une chambre de vide en forme de cupule de 5 cm de diamètre et de 2,5 cm de hauteur avec une base en polyuréthane de qualité médicale relativement souple donc peu traumatisante pour le scalp fœtal, un lac de traction ajustable et une poignée ergonomique. Les surfaces, extérieure et intérieure, de la cupule sont lisses pour ne pas traumatiser l'enfant ou la filière génitale de la patiente.

Sur le dôme de la cupule apparaît une encoche spécialement conçue pour le doigt de l'opérateur et lui permettre de s'orienter lors de l'insertion et pendant la progression fœtale

L'aspiration à l'intérieur de la cupule est circonférentielle au toit interne pour éliminer tout risque de bouchon. La surface de cette paroi est lisse et légèrement convexe.

Le lac de traction, formé d'un câble métallique recouvert d'une protection plastique portant des marques de repérage à 5 cm et 10 cm, est situé exactement au centre du dôme de la cupule avec une mobilité sur 360°. Sa longueur totale de 60 cm est modifiable selon les besoins de l'opérateur en insérant l'extrémité dans les taquets coinceurs de la poignée.

Le tuyau souple d'aspiration qui part du dôme de la cupule d'une longueur de 1,40 mètre (pour laisser toute liberté de mouvements à l'opérateur) se termine par un adaptateur à diamètre variable conçu pour se connecter à la majorité des pompes à vide disponibles. Ce tuyau est renforcé par un tube armé intérieur à la sortie de la cupule pour éliminer tout risque d'obstruction lors d'un pliage durant l'extraction.

15.2. Indications

Les principales indications d'application de la ventouse obstétricale iCup sont les suivantes :

- Arrêt de progression de la tête fœtale à partir du détroit moyen de l'excavation, surtout s'il existe un défaut de flexion. Au niveau du détroit moyen, la ventouse obstétricale semble être le meilleur instrument du fait de son action de flexion. Les arrêts de la descente d'une présentation céphalique au niveau du détroit moyen sont très souvent des défauts de rotation d'une position postérieure et le complément de flexion permet la rotation vers une position antérieure et l'accouchement par voie basse
- Aide à l'expulsion pour fatigue ou agitation maternelle : en accentuant la flexion, la ventouse obstétricale facilite la fixation sous-symphysaire de l'occiput

- Inertie utérine en complément des ocytociques
- Toutes les anomalies du rythme cardiaque fœtal (RCF) justifiant une extraction instrumentale
- Aide à l'expulsion chez les femmes porteuses d'une cicatrice utérine
- Aide à l'expulsion pour maladie maternelle (éclampsie, pré-éclampsie, cardiopathie, insuffisance respiratoire, para ou tétraplégie, anévrisme cérébral, rétinopathie, …).

15.3. Contre-indications

Comme pour les autres ventouses obstétricales, les principales contre-indications à l'application de la ventouse obstétricale iCup sont : la prématurité avant 34 semaines, les présentations de la face, du siège, du front et transversales (épaules), une dilatation cervicale incomplète, une présentation non-engagée ou à partie haute de l'excavation, une variété de position indéterminée, la présence d'une importante bosse séro-sanguine (les bosses séro-sanguines modérées souvent observées quand le travail a été long ne sont pas une contre-indication), les syndromes hémorragiques du nouveau-né (hémophilie, trouble de la crase sanguine in utero, prise continue de phénobarbital par la mère), les fœtus avec un trouble de la minéralisation osseuse (ostéogénèse imparfaite), les cals du bassin, la disproportion fœto-pelvienne, la suspicion de macrosomie fœtale, une anesthésie générale, une mère non-coopérante, un opérateur inexpérimenté.

15.4. Mode opératoire

Les conditions préalables à l'application de la ventouse obstétricale doivent respecter les critères suivants : Indication d'extraction bien posée, possibilité de faire une césarienne rapidement en cas d'échec. Il est indispensable de pouvoir réaliser les extractions instrumentales fœtales dans une salle proche de la salle de césarienne, voir dans cette dite salle.

Lorsqu'une extraction est indiquée, l'obstétricien doit prendre quelques instants pour expliquer la situation aux parents. L'adhésion et la participation de la parturiente à une extraction fœtale par ventouse sont indispensables afin d'obtenir des efforts expulsifs efficaces lors des tractions.

L'asepsie doit être parfaite.

Un sondage évacuateur avant la pose de l'instrument sera réalisé si nécessaire

Une anesthésie péridurale est souhaitable. Une anesthésie générale est inutile et gênante : la participation de la patiente par ses efforts de poussée est essentielle, car les contractions utérines associées aux efforts de poussée assurent 60 % de la force totale permettant l'accouchement.

Les membranes doivent être rompues.

La présentation est céphalique, le col à dilatation complète.

Le diagnostic d'orientation, du degré de déflexion et d'asynclitisme de la tête fœtale doivent être parfaitement connus afin de poser correctement l'instrument. La cupule doit s'appliquer le plus près possible de l'occiput. En effet, plus long est le bras de levier (distance entre l'articulation atloïdo-occipitale et l'occiput) plus aisée sera la flexion de la tête et plus faible sera la force de traction pour obtenir cette flexion. Une erreur de diagnostic fera de la ventouse un instrument de déflexion à l'origine d'une dystocie. Le toucher vaginal, par la recherche des fontanelles, des sutures voire des oreilles peut permettre le diagnostic de présentation. La palpation

abdominale, avec la localisation des fesses et du dos fœtal, indique le côté de l'occiput. En cas de doute persistant (bosse séro-sanguine, asynclitisme), s'aider de l'échographie en salle de travail. La localisation du dos fœtal permet de dire si la présentation est droite ou gauche. La visualisation aisée d'au moins un globe oculaire affirme la variété postérieure et en cas de non visualisation des globes oculaires, la variété antérieure. S'aider aussi des rapports anatomiques normaux (cervelet, thalamus, ...)

Le diagnostic de la hauteur de la présentation parfaitement établi. L'engagement de la présentation fœtale au niveau de la partie moyenne de l'excavation est nécessaire (figure 2) [1] .

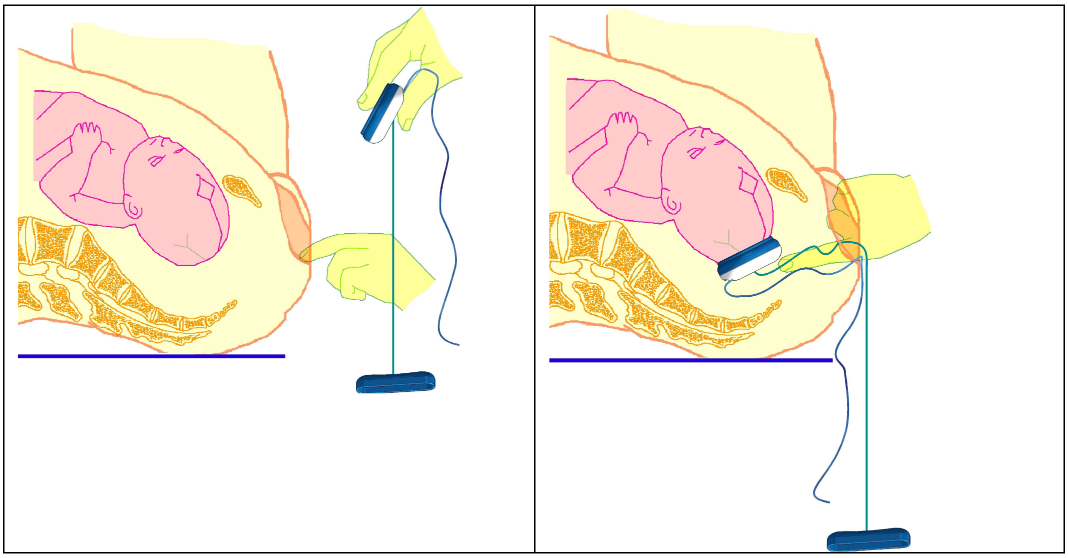

Figure 3 : mise en place de la cupule iCup

DATES ET SIGNATURES

Ce protocole a été lu et approuvé à la date notée en en tête

INVESTIGATEUR COORDONNATEUR

Dr Véronique EQUY

Pôle Couple-Enfant – Service de chirurgie obstétrique

CHU de Grenoble

DATE : |2|7|/|0|5|/|2|0|1|1|

SIGNATURE :

POUR LE PROMOTEUR

Mme Hélène SABBAH-GUILLAUME, Directrice de la Recherche et des Partenariats

Délégation à la Recherche Clinique et à l'Innovation

DATE : |2|7|/|0|5|/|2|0|1|1|

SIGNATURE :

16. RÉFÉRENCES BIBLIOGRAPHIQUES

1. *Schaal JP, Riethmuller D, Menget A. Extractions instrumentales du fœtus : ventouse obstétricale. Encycl Méd Chir Paris: Elsevier, 2004 (vol Obstétrique).*
2. *Bergsjo P, Schmidt E, Pusch D. Differences in the reported frequencies of some obstetrical interventions in Europe. Br J Obstet Gynaecol 1983;90:628-32.*
3. *Hillier CE, Johanson RB. Worldwide survey of assisted vaginal delivery. Int J Gynaecol Obstet 1994;47:109-14.*
4. *Ott WJ. Vacuum extraction. Obstet Gynecol Surv 1975;30:643-9.*
5. *Chalmers JA, Chalmers I. The obstetric vacuum extractor is the instrument of first choice for operative vaginal delivery. Br J Obstet Gynaecol 1989;96:505-6.*
6. *Ventura SJ, Martin JA, Curtin SC, Mathews TJ. Report of final natality statistics, 1996. Mon Vital Stat Rep 1998;46:1-99.*
7. *Learman LA. Regional differences in operative obstetrics: a look to the South. Obstet Gynecol 1998;92:514-9.*
8. *Miksovsky P, Watson WJ. Obstetric vacuum extraction: state of the art in the new millennium. Obstet Gynecol Surv 2001;56:736-51.*
9. *Hamilton BE, Martin JA, Ventura SJ. Births: preliminary data for 2005. Natl Vital Stat Rep 2006;55:1-18.*
10. *Martin JA, Hamilton BE, Sutton PD, Ventura SJ, Menacker F, Kirmeyer S. Births: final data for 2004. Natl Vital Stat Rep 2006;55:1-101.*
11. *Martin JA, Hamilton BE, Sutton PD, Ventura SJ, Menacker F, Kirmeyer S, Munson ML. Births: final data for 2005. Natl Vital Stat Rep 2007;56:1-103.*
12. *Curtin SC. Recent changes in birth attendant, place of birth, and the use of obstetric interventions, United States, 1989-1997. J Nurse Midwifery 1999;44:349-54.*
13. *Menacker F, Martin JA. Expanded health data from the new birth certificate, 2005. Natl Vital Stat Rep 2008;56:1-24.*
14. *Macfarlane A. At last--maternity statistics for England. Bmj 1998;316:566-7.*
15. *O'Connell MP, Lindow SW. Trends in obstetric care in the United Kingdom. J Obstet Gynaecol 2000;20:592-3.*
16. *Richardson A, Mmata C. NHS Maternity Statistics, England: 2005-06. 2007. http://www.ic.nhs.uk/.*
17. *Santé Canada. Rapport sur la santé périnatale au Canada 2003. Ottawa (Ont.): ministre des Travaux publics et des Services gouvernementaux, 2003.*
18. *Roberts CL, Algert CS, Carnegie M, Peat B. Operative delivery during labour: trends and predictive factors. Paediatr Perinat Epidemiol 2002;16:115-23.*
19. *Roberts CL, Algert CS, Douglas I, Tracy SK, Peat B. Trends in labour and birth interventions among low-risk women in New South Wales. Aust N Z J Obstet Gynaecol 2002;42:176-81.*
20. *Laws PJ, Abeywardana S, Grayson N, Sullivan EA. Australia's Mothers and Babies 2005. Perinatal statistics series no.20. AIWH cat. no. PER 40. 2007. http://www.npsu.unsw.edu.au/NPSUweb.nsf/page/ps20.*
21. *Schaal JP, Rabenja CA, Gay C, Riethmuller D, Maillet R. Extractions instrumentales et césariennes dans les CHU français en 1993XXV assises nationales des sages-femmes. Besançon, 1997.*
22. *L'expression abdominale durant la deuxième phase de l'accouchement. 2007. http://www.has-sante.fr/portail/jcms/c_513170/l-expression-abdominale-durant-la-2e-phase-de-l-accouchement.*
23. *Attilakos G, Sibanda T, Winter C, Johnson N, Draycott T. A randomised controlled trial of a new handheld vacuum extraction device. Bjog 2005;112:1510-5.*
24. *Attilakos G, Sibanda T, Winter C, Johnson N, Draycott T. A randomised trial of a new handheld vacuum extraction device. Bjog 2006;113:494-5.*

25. *Groom KM, Jones BA, Miller N, Paterson-Brown S. A prospective randomised controlled trial of the Kiwi Omnicup versus conventional ventouse cups for vacuum-assisted vaginal delivery. Bjog 2006;113:183-9.*
26. *Johanson R, Menon V. Soft versus rigid vacuum extractor cups for assisted vaginal delivery. Cochrane Database Syst Rev 2000:CD000446.*
27. *Schaal JP, Equy V, Hoffmann P. Recommandations pour la pratique clinique : Extractions instrumentales. Comparaison ventouse forceps. J Gynecol Obstet Biol Reprod (Paris) 2008;37:231-43.*
28. *Riethmuller D, Ramanah R, Maillet R, Schaal JP. Recommandations pour la pratique clinique : Extractions instrumentales. Ventouses : descriptions, mécanique, indications et contre-indications. J Gynecol Obstet Biol Reprod (Paris) 2008;37:210-21.*
29. *Perard L, Hot A, Cucherat M, Simon M, Desmurs H, Coppere B, Girard-Madoux MH, Boissel JP, Ninet J. Essais de non-infériorité dans la maladie thromboembolique veineuse. Une lecture critique est nécessaire ! Rev Med Interne 2007;28:731-6.*
30. *Payen JF, Chanques G, Mantz J, Hercule C, Auriant I, Leguillou JL, Binhas M, Genty C, Rolland C, Bosson JL. Current practices in sedation and analgesia for mechanically ventilated critically ill patients: a prospective multicenter patient-based study. Anesthesiology 2007;106:687-95; quiz 891-2.*
31. *Machin D, Campbell MJ. Statistical Tables for Design of Clinical Trials. Oxford: Blackwell Scientific Publications, 1987.*
32. *Fleiss JL, Tytun A, Ury SHK. A simple approximation for calculating sample sizes for comparing independent proportions. Biometrics 1980;36:343-6.*
33. *Schaal JP. Recommandations pour la pratique clinique : modalités de surveillance fœtale pendant le travail. Introduction. J Gynecol Obstet Biol Reprod (Paris) 2008;37 Suppl 1:S3-4.*

17. ANNEXES

17.1. ANNEXE A: FORMULAIRE D'INFORMATION A LA PATIENTE

Document constitué en application du Code de Santé Publique.

Formulaire d'information à la patiente pour l'Evaluation du rapport bénéfice/risque d'une nouvelle ventouse obstétricale à usage unique versus la ventouse obstétricale de référence

Document constitué en application du Code de Santé Publique.

Médecin investigateur coordonnateur : Dr Véronique EQUY (en remplacement du Pr Jean-Patrick Schaal)
Clinique Universitaire de Gynécologie Obstétrique - Pôle Couple - Enfant
CHU de Grenoble BP 217 38043 Grenoble Cedex 09
Tel. : 04-76-76-94-50 - Fax : 04-76-76-94-51

Promoteur : CHU de Grenoble - Direction de la Recherche Clinique et de l'Innovation
CHU de Grenoble, 38043 Grenoble cedex 09
Tel : 04 76 76 59 57 - Fax : 04 76 76 52 21

Madame,

Le Dr……………………………….vous propose de participer à une recherche biomédicale. Elle est organisée par le CHU de GRENOBLE. Elle concerne l'utilisation d'une nouvelle ventouse obstétricale.

Vous êtes libre d'accepter ou de refuser de participer à cette recherche. Vous pouvez retirer à tout moment votre consentement sans encourir aucune responsabilité ni aucun préjudice de ce fait.

Un délai de réflexion vous sera accordé avant la signature du consentement, si vous le désirez.

Lors de l'accouchement, seules les patientes, qui auront recours à la ventouse, seront concernées par cette étude.

Afin d'éclairer votre décision, voici des informations concernant cette recherche :

TITRE IDENTIFIANT LA RECHERCHE

"Evaluation du rapport bénéfice/risque d'une nouvelle ventouse obstétricale à usage unique versus la ventouse obstétricale de référence"

BUT DE L'ETUDE

Le but de cette étude est d'évaluer l'efficacité d'une nouvelle ventouse obstétricale à usage unique (iCup).

DISPOSITIF A L'ETUDE

La ventouse iCup à l'étude est une nouvelle ventouse fabriquée par la Société Collin. Contrairement aux ventouses obstétricales actuelles, elle a l'avantage d'être à usage unique. Elle présente aussi les mêmes caractéristiques que les ventouses métalliques stérilisables.

La prise en charge de votre accouchement ne sera pas modifiée par votre participation à l'étude. Seule la ventouse utilisée diffèrera, si ce recours est nécessaire. Lors de l'accouchement, seules les patientes, qui auront recours à la ventouse, seront concernées par cette étude.

Si vous ne souhaitez pas participer à l'étude, vous bénéficierez de la prise en charge classique adaptée à votre situation. C'est l'utilisation de la ventouse métallique stérilisable, dans le cas où l'utilisation d'une ventouse soit indiquée.

METHODOLOGIE

Il s'agit d'une étude d'efficacité, randomisée (tirage au sort de la ventouse utilisée), comparative entre la ventouse habituelle et la ventouse iCup. Elle est réalisée en ouvert (vous et votre médecin savez quelle ventouse est utilisée) et est multicentrique (6 centres, 700 femmes).

DEROULEMENT DE L'ETUDE

Si vous acceptez de participer à l'étude, vous devez signer le formulaire de consentement ci-joint. Si l'obstétricien juge utile l'emploi d'une ventouse au cours du travail, vous serez intégrée dans l'un des deux groupes : ventouse iCup versus ventouse habituelle. Les paramètres recueillis le jour de l'accouchement seront les suivants : Caractéristiques maternelles, Caractéristiques du travail, Indication et caractéristiques de l'extraction instrumentale, Caractéristiques fœtales, Etat maternel et données néonatales.

Un examen médical de l'enfant et de la mère sera effectué pendant le séjour en maternité. En pratique habituelle, un examen est fait à JO et à la sortie de l'enfant. Un examen sera pratiqué quotidiennement si la santé de la mère (en cas d'épisiotomie par exemple) ou/et de l'enfant le nécessite.

DUREE DE L'ETUDE

Vous serez incluse dans l'étude uniquement pendant votre séjour à la maternité.

Vous pourrez vous prêter à une autre étude clinique durant votre participation à l'étude iCup et toute de suite après la fin de cette étude.

BENEFICES

Bénéfices attendus : cette ventouse pourrait se révéler aussi efficace et moins traumatique pour l'enfant que les ventouses utilisées classiquement.

CONTRAINTES ET RISQUES

Les contraintes et les risques prévisibles sont ceux liés à l'utilisation de toute ventouse.

Ils vous ont été exposés par votre obstétricien indépendamment de cette étude.

Effets indésirables :

1/ <u>Atteintes maternelles</u>

La ventouse obstétricale s'applique sur le crâne fœtal et n'augmente pas ses dimensions. Elle évite les problèmes inhérents à une anesthésie générale. La majorité des études ont attribué à la ventouse moins de conséquences délétères pour les parties molles de la femme que le forceps. Les lésions rencontrées sont celles entraînées par un mauvais contrôle du dégagement ou par une mauvaise vérification de la mise en place du dispositif.

Atteintes vaginales, vulvaires et périnéales

Atteintes rares : périnés complets ou compliqués, lésions cervicales, hémorragies

Atteintes évitables : atteintes de la muqueuse vaginale dont un repli était resté pincé entre la cupule et la présentation, atteintes cervicales (si la ventouse a été posée avant la dilatation cervicale complète) qui relevait du même mécanisme.

Atteintes exceptionnelles : détachement annulaire du col, fistules vésico-vaginale ou vésico-utérine

Atteintes à distance : incontinence anale et/ou urinaire

2/ Atteintes néonatales

Atteintes du sclap fœtal : bosse séro-sanguine, excoriation du cuir chevelu, décollement cutané localisé, céphalhématome ou hématome sous-périosité, hématome sous cutané du cuir chevelu

Autres complications : hémorragies cérébro-méningées, hémorragies rétiniennes, fractures du crâne, ictère, infections, lésions neurologiques

PROTECTION DES PERSONNES

Cette recherche biomédicale a reçu un avis favorable du Comité de Protection des Personnes Sud-Est V le 29/07/2009 et a été autorisée par l'Agence française de sécurité sanitaire des produits de santé le 01/07/2009.

Le CHU de Grenoble a pris toutes les dispositions prévues par la loi sur la protection des personnes (contrat d'assurance SHAM n°126.959; 18, rue Edouard Rochet, 69372 Lyon Cedex 08).

Un exemplaire de cette fiche d'information vous est destiné.

A l'issue de cette recherche vous pourrez être informé de ses résultats globaux par courrier postal.

CONFIDENTIALITE DES DONNEES VOUS CONCERNANT

Dans le cadre de la recherche biomédicale à laquelle le CHU de Grenoble vous propose de participer, un traitement de vos données personnelles va être mis en œuvre pour permettre d'analyser les résultats de la recherche au regard de l'objectif de cette dernière. A cette fin, les données médicales vous concernant, seront transmises au Promoteur de la recherche ou aux personnes ou sociétés agissant pour son compte, en France ou à l'étranger. Ces données seront identifiées par un numéro de code et/ou vos initiales ou les deux premières lettres de votre nom et la première lettre de votre prénom. Ces données pourront également, dans des conditions assurant leur confidentialité, être transmises aux autorités de santé françaises ou étrangères et à d'autres entités du promoteur. Conformément aux dispositions de loi relative à l'informatique aux fichiers et aux libertés du 6 janvier 1978, vous disposez d'un droit d'accès et de rectification. Vous disposez également d'un

droit d'opposition à la transmission des données couvertes par le secret professionnel susceptibles d'être utilisées dans le cadre de cette recherche et d'être traitées.

Vous pouvez également accéder directement ou par l'intermédiaire d'un médecin de votre choix à l'ensemble de vos données médicales en application des dispositions de l'article L 1111-7 du Code de la Santé Publique. Ces droits s'exercent auprès du médecin qui vous suit dans le cadre de la recherche et qui connaît votre identité.

FRAIS

Aucun frais ne vous sera facturé du fait de votre participation à l'étude.

Nous vous remercions pour votre participation à cette étude et pour les bénéfices que celle-ci pourra apporter à l'ensemble des femmes enceintes pour lesquelles il existe à l'accouchement une indication d'extraction instrumentale.

17.2. ANNEXE B: CONSENTEMENT DE PARTICIPATION

CONSENTEMENT DE PARTICIPATION	
**Titre identifiant la recherche : Evaluation du rapport bénéfice/risque d'une nouvelle ventouse obstétricale à usage unique versus la ventouse obstétricale de référence**	
**Médecin investigateur responsable de l'étude :**<br>Dr Véronique EQUY (en remplacement du Pr Jean-Patrick Schaal)<br>**Coordonnées :**<br>Clinique Universitaire de Gynécologie Obstétrique<br>Pôle Couple - Enfant<br>CHU de Grenoble BP 217 - 38043 Grenoble Cedex 09<br>Tel. : 04-76-76-94-50 - Fax : 04-76-76-94-51	**Promoteur :**<br>CHU de Grenoble<br>**Coordonnées :**<br>Direction de la Recherche Clinique et de l'Innovation<br>CHU de Grenoble<br>38043 Grenoble cedex 09<br>Tel : 04 76 76 59 57 - Fax : 04 76 76 52 21

J'ACCEPTE DE PARTICIPER A CETTE RECHERCHE DANS LES CONDITIONS PRECISEES CI-DESSUS.

Si je le désire, j'ai le droit de refuser de participer à cette recherche. Je peux aussi retirer mon consentement à tout moment sans encourir aucune responsabilité ni aucun préjudice de ce fait. J'en informerai alors le Dr…………………………………….……….

Les données qui me concernent resteront strictement confidentielles. Je n'autorise leur consultation que par des personnes soumises au secret professionnel et collaborant à cette recherche.

Je pourrai à tout moment demander toute information complémentaire auprès des médecins investigateurs.

J'accepte que les données enregistrées à l'occasion de cette étude puissent faire l'objet d'un traitement informatisé, après l'anonymat, par le promoteur ou pour son compte. J'ai bien noté que mon droit d'accès prévu par la loi informatique et liberté s'exerce à tout moment.

J'ai reçu de l'investigateur une fiche d'information détaillée et obtenu une réponse à l'ensemble de mes questions. Je reconnais avoir bénéficié d'un délai de réflexion suffisant. J'ai reçu une copie du présent document. J'ai été informée qu'une copie sera également conservée par les organisateurs dans des conditions garantissant la confidentialité. J'y consens. J'ai été informée que conformément à la réglementation sur les recherches biomédicales, le Comité de Protection des Personnes Sud-Est V a donné un avis favorable le 29/07/2009 et l'Agence française de sécurité sanitaire des produits de santé a donné son autorisation le 01/07/2009, pour la réalisation de cette recherche.

Nom de la volontaire :	Nom de l'investigateur :
____	____________________________________
Date :	Date :
Signature de la volontaire	Signature de l'investigateur

17.3. ANNEXE C : BUDGET

PHRC 2009 - Appel à projets national - Détail de la demande financière					
**Etablissement promoteur :** **CHU de Grenoble**	**Nom de l'investigateur principal :** **Pr Jean-Patrick Schaal**			**Projet n° :**	
NATURE DE LA DEPENSE	**DETAIL**	**2009**	**2010**	**2011**	**Total en K€**
**DEPENSES DE PERSONNEL**					
**Personnel médical et non médical**					
Collaboration Cellule Innovation / CIC : ARC coordonateur (monitoring des données, aide ponctuelle locale, ce qui équivaut à un mi temps annuel)		20	20		40
ARCs terrain sur site 1/8 temps pendant un an 5 000 × 6 centres		27			27
Collaboration Cellule Innovation / CIC : médecin épidémiologiste et statisticien (mise en place du protocole, nettoyage de la base de données, analyse statistique)		3	10		13
Frais de traduction scientifique			2		2
Sous-total (1)					**82**
**DEPENSES MEDICALES**					
Matériel biomédical : ventouse obstétricale iCup					Fourni
Sous-total (2)					**0**
**DEPENSES HOTELIERES ET GENERALES**					
Cahier d'observation électronique		20			20
Liste de randomisation		1			1
Fournitures de bureau et informatique		0,5			0,5
Déplacement / missions ARC coordonateur sur les 5 centres		8,5	5		13.5
Déplacement investigateurs (réunions de début et fin d'étude, et congrès)		5	9		14
Sous-total (3)					**49**
**DEPENSES FINANCIERES**					
Frais financiers (mat. médical)					
Charges exceptionnelles					
Amortissements					
Frais de gestion	DRCI 5%	7			7
Sous-total (4)					**7**
**TOTAL (1)+(2)+(3)+(4)**					**138,00**

17.4. ANNEXE D : NOTICE ICUP

COLLIN 3 rue de Robinson 92227 BAGNEUX Cedex info@collinmedical.fr
Tél. : 01 49 08 08 88 Fax : 01 49 08 08 89 www.collinmedical.fr

iCup®

VENTOUSE OBSTETRICALE STERILE A USAGE UNIQUE
(conçue par le Pr. JP. Schaal - CHU Grenoble)(Brevet : en cours)
Modèle JL7000 Diamètre : 5 cm

NOTICE D'INFORMATION POUR L'UTILISATEUR

Lire attentivement toutes les informations avant utilisation

Contenu

Une ventouse obstétricale *iCup* avec lac de traction, poignée ergonomique et tube de connexion à pompe à vide

Description

La ventouse obstétricale *iCup* est un dispositif d'extraction instrumentale du fœtus. *Elle est stérile à usage unique prête à l'emploi* dans les conditions d'utilisation et pour les indications recommandées.

La ventouse *iCup* comprend une chambre de vide en forme de cupule de 5 cm de diamètre et de 2,5 cm de hauteur avec une base en polyuréthane de qualité médicale relativement souple donc peu traumatisante pour le scalp fœtal, un lac de traction ajustable et une poignée ergonomique. Les surfaces, extérieure et intérieure, de la cupule sont lisses pour ne pas traumatiser l'enfant ou la filière génitale de la patiente.

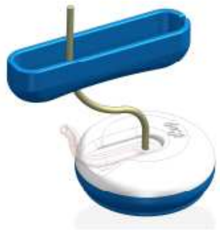

Sur le dôme de la cupule apparaît une encoche spécialement conçue pour le doigt de l'opérateur et lui permettre de s'orienter lors de l'insertion et pendant la progression fœtale

L'aspiration à l'intérieur de la cupule est circonférentielle au toit interne pour éliminer tout risque de bouchon. La surface de cette paroi est lisse et légèrement convexe.

Le lac de traction, formé d'un câble métallique recouvert d'une protection plastique portant des marques de repérage à 5 cm et 10 cm, est situé exactement au centre du dôme de la cupule avec une mobilité sur 360°. Sa longueur totale de 60 cm est modifiable selon les besoins de l'opérateur en insérant l'extrémité dans les taquets coinceurs de la poignée.

Le tuyau souple d'aspiration qui part du dôme de la cupule d'une longueur de 1,40 mètres (pour laisser toute liberté de mouvements à l'opérateur) se termine par un adaptateur à diamètre variable conçu pour se connecter à la majorité des pompes à vide disponibles. Ce tuyau est renforcé par un tube armé intérieur à la sortie de la cupule pour éliminer tout risque d'obstruction lors d'un pliage durant l'extraction.

Indications

Les principales indications d'application de la ventouse obstétricale *iCup* sont les suivantes :

- Arrêt de progression de la tête fœtale à partir du détroit moyen de l'excavation, surtout s'il existe un défaut de flexion. Au niveau du détroit moyen, la ventouse obstétricale semble être le meilleur instrument du fait de son action de flexion. Les arrêts de la descente d'une présentation céphalique au niveau du détroit moyen sont très souvent des défauts de rotation d'une position postérieure et le complément de flexion permet la rotation vers une position antérieure et l'accouchement par voie basse
- Aide à l'expulsion pour fatigue maternelle : en accentuant la flexion, la ventouse obstétricale facilite la fixation sous-symphysaire de l'occiput
- Inertie utérine en complément des ocytociques
- Toutes les anomalies du rythme cardiaque fœtal (RCF) justifiant une extraction instrumentale
- Aide à l'expulsion chez les femmes porteuses d'une cicatrice utérine
- Aide à l'expulsion pour maladie maternelle (éclampsie, prééclampsie, cardiopathie, insuffisance respiratoire, para ou tétraplégie, anévrisme cérébral, rétinopathie, …).

REMARQUE : Il faut toujours tenir compte du bien être fœtal et de l'estimation échographique du poids fœtal avant de poser une indication d'extraction. Des anomalies du rythme cardiaque et/ou une estimation de poids fœtal supérieure à la normale doivent faire reconsidérer l'indication d'extraction fœtale en fonction du calcul risques/bénéfices.

Contre-indications

Les principales contre-indications à l'application de la ventouse obstétricale *iCup* sont :

- La prématurité avant 34 semaines
- Les présentations de la face, du siège, du front et transversales (épaules)
- Une dilatation cervicale incomplète
- Une présentation non-engagée ou à partie haute de l'excavation
- Une variété de position indéterminée
- La présence d'une importante bosse séro-sanguine (les bosses séro-sanguines modérées souvent observées quand le travail a été long ne sont pas une contre-indication)
- Les syndromes hémorragiques du nouveau-né (hémophilie, trouble de la crase sanguine in utero, prise continue de phénobarbital par la mère)
- Les fœtus avec un trouble de la minéralisation osseuse (ostéogénèse imparfaite)
- Les cals du bassin
- La disproportion fœto-pelvienne
- La suspicion de macrosomie fœtale
- Une anesthésie générale
- Une mère non-coopérante.
- Un opérateur inexpérimenté

NB : la présence d'une scalp-électrode ou les micro-prises de sang au scalp ne sont pas considérées comme des contre-indications à la ventouse obstétricale

Mode d'utilisation : pré-requis

La ventouse *iCup* est un instrument de flexion, de traction limitée et de rotation induite. Elle permet d'aider la femme à enfanter par les voies naturelles

Les conditions préalables à l'application de la ventouse obstétricale doivent respecter les critères suivants, critères qui ne doivent être considérés comme des recommandations en raison des différences entre chaque accouchement et des différences de technique propre à chaque médecin :

- Indication d'extraction bien posée
- Possibilité de faire une césarienne rapidement en cas d'échec. Il est indispensable de pouvoir réaliser les extractions instrumentales fœtales dans une salle proche de la salle de césarienne, voir dans cette dite salle.
- Lorsqu'une extraction est indiquée, l'obstétricien doit prendre quelques instants pour expliquer la situation aux parents. L'adhésion et la participation de la parturiente à une extraction fœtale par ventouse sont indispensables afin d'obtenir des efforts expulsifs efficaces lors des tractions

- Asepsie parfaite
- Vessie vide. Un sondage évacuateur avant la pose de l'instrument sera réalisé si nécessaire.
- Membranes rompues.
- Présentation céphalique
- Col à dilatation complète
- Diagnostic d'orientation, du degré de déflexion et d'asynclitisme de la tête fœtale parfaitement connus afin de poser correctement l'instrument. La cupule doit s'appliquer le plus près possible de l'occiput. En effet, plus long est le bras de levier (distance entre l'articulation atloïdo-occipitale et l'occiput) plus aisée sera la flexion de la tête et plus faible sera la force de traction pour obtenir cette flexion. Une erreur de diagnostic fera de la ventouse un instrument de déflexion à l'origine d'une dystocie. Le toucher vaginal, par la recherche des fontanelles, des sutures voire des oreilles peut permettre le diagnostic de présentation. La palpation abdominale, avec la localisation des fesses et du dos fœtal, indique le côté de l'occiput. En cas de doute persistant (bosse séro-sanguine, asynclitisme), s'aider de l'échographie en salle de travail. La localisation du dos fœtal permet de dire si la présentation est droite ou gauche. La visualisation aisée d'au moins un globe oculaire affirme la variété postérieure et en cas de non visualisation des globes oculaires, la variété antérieure. S'aider aussi des rapports anatomiques normaux (cervelet, thalamus...)
- Diagnostic de la hauteur de la présentation parfaitement établi. L'engagement de la présentation fœtale en partie moyenne est nécessaire

Procédure d'utilisation

- Identifier le dispositif sur son étiquetage. Vérifier l'intégrité de l'emballage et la date de péremption (cf. explication des idéogrammes en annexe)
- Sortir la ventouse et sa poignée du sachet stérile
- Adapter la poignée au lac de traction à la longueur désirée
- Raccorder le tube d'aspiration de la ventouse en introduisant son raccord universel dans le tuyau de la pompe à vide
- Mettre en place la ventouse sur le scalp fœtal, le plus près possible de l'occiput fœtal
- Faire un premier palier de dépression jusqu'à 200mbar. Vérifier qu'il n'y a pas de tissus mous maternels entre la présentation et la ventouse
- Passer au deuxième palier de dépression de 800 mbar. En général, une dépression de 0,6 à 0,7 kg est suffisante. Ne jamais dépasser 0,8 kg.
- Suivre le mode opératoire recommandé

Précautions lors de l'utilisation de la pompe à dépression

Choix d'un appareil d'aspiration compatible :

.

- *NE PAS UTILISER LE VIDE MURAL DU BLOC OPERATOIRE OU DE LA SALLE D'ACCOUCHEMENT*
- La pompe doit produire la dépression nécessaire à l'aide à l'extraction du fœtus par ventouse de façon contrôlée grâce à un affichage lisible indiquant les mesures actuelles en mbar, mmHg ou autres unités (cf. tableau d'équivalence des unités de dépression en annexe)
- Les spécifications de la pompe doivent être comprises dans les limites suivantes :
  Vide niveau de mer :-900 mbar
  Capacité d'aspiration : +/- 2 l/min
  Marquage CE
  Normes : ISO 13485-2003, ISO 60 601-1, ISO 10079-1
- Vérifier l'appareil et la stabilité de l'affichage avant chaque utilisation. L'indication manométrique doit croître à partie du déclenchement du vide et rester stable lorsque la pompe est arrêtée par l'opérateur au niveau de dépression désiré
- En cas de fuite, vérifier l'intégrité des tuyaux de la ventouse et de la pompe et que la connexion entre la pompe et le tube de vide de la ventouse est étanche
- La responsabilité du bon fonctionnement de la pompe incombe au service biomédical de maintenance de l'établissement
- *Il est recommandé d'utiliser une pompe à contrôle automatique du vide*, seule garantie de ne pas subir de petites pertes progressives de vide qui peuvent provoquer un lâchage de la ventouse*

*telle que le modèle ATMOS 350 A auquel a succédé ATMOS 351 Natal. Ces deux appareils d'aspiration automatiques ont été conçus spécifiquement pour l'utilisation avec une ventouse obstétricale car ils offrent la présélection des deux paliers à 200 et 800mbar et un retour progressif à zéro du vide dans la ventouse

Précautions Opératoires

Lire attentivement le MODE OPERATOIRE de la ventouse iCup

L'application de la ventouse obstétricale ***iCup*** requiert une parfaite connaissance de la mécanique obstétricale, une formation professionnelle spécialisée et des conditions d'asepsie adaptées

Une position correcte de la cupule ***iCup*** sur la tête fœtale est garante du succès de la manœuvre. La cupule doit être appliquée au plus près du « point de flexion ». Le « point de flexion » est situé sur la suture sagittale 3 cm en avant de la petite fontanelle à l'intersection entre le diamètre sincipito-mentonnier et le scalp fœtal. En pratique, il faut pousser la ventouse ***iCup*** le plus loin possible vers l'occiput fœtal

Quand faut-il renoncer ?

- ***Efforts maternels inefficaces après 10 minutes d'utilisation dans les conditions recommandées***
- ***Après une période de temps correspondant à cinq contractions utérines***
- ***Après deux lâchages de la ventouse***

Effets indésirables

Causes des échecs et des complications de la ventouse obstétricale

- Déflexion ou asynclitisme trop important de la tête fœtale
- Rétraction du col
- Dilatation incomplète du col
- Équipement défectueux, absence de matériels adéquats
- Lâchage lors d'une traction dans un mauvais axe ou en dehors des efforts expulsifs maternels
- Mauvais positionnement de la ventouse
- Mère non-coopérante
- Anesthésie générale
- Disproportion fœto-pelvienne
- Lâchage du à une dépression défectueuse ou non soutenue

Atteintes maternelles :

La ventouse obstétricale s'applique sur le crâne fœtal et n'augmente pas ses dimensions. Elle évite les problèmes inhérents à une anesthésie générale. La majorité des études ont attribué à la ventouse moins de conséquences délétères pour les parties molles de la femme que le forceps. Les lésions rencontrées sont celles entraînées par un mauvais contrôle du dégagement ou par une mauvaise vérification de la mise en place du dispositif

- **Atteintes vaginales, vulvaires et périnéales**
- **Atteintes rares**
  Périnées complets ou compliqués, lésions cervicales, hémorragies
- **Atteintes évitables**
  Atteintes de la muqueuse vaginale dont un repli était resté pincé entre la cupule et la présentation, atteintes cervicales (si la ventouse a été posée avant la dilatation cervicale complète) qui relevait du même mécanisme.
- **Atteintes exceptionnelles**
  Détachement annulaire du col, fistules vésico-vaginale ou vésico-utérine
- **Atteintes à distance**
  Incontinences anale et/ou urinaire

Atteintes néonatales :

- **Atteintes du scalp fœtal**
  Bosse séro-sanguine, excoriation du cuir chevelu, décollement cutané localisé, céphalhématome ou hématome sous-périosté, hématome sous-cutané diffus du cuir chevelu

- **Autres complications**
  Hémorragies cérébro-méningées, hémorragies rétiniennes, fractures du crâne, ictère, infections, lésions neurologiques

Référence bibliographique

Schaal JP, Riethmuller D, Maillet R, Uzan M Mécanique et Techniques Obstétricales-Ventouse Obstétricale Edit. Sauramps Médical p.675-714 (2007)

Pour toute demande de renseignement sur la ventouse *iCup* s'adresser à :

COLLIN
5 rue Robinson
92227 BAGNEUX Cedex
Service Clients : 01 49 08 08 88

CE 0459

©2007 COLLIN Fabriqué en France

16C0703Cbfg

5/6

TABLEAU DE CONVERSION DES MESURES DE DEPRESSION

mbar	mmHg	Kg/cm2	cmH20	KPa	Inches Hg	Lb/In2
**200***	150	**0,15**	204	**20**	5.9	2.9
400	300	0,30	408	40	11.8	5.8
600	450	0,45	612	60	17.7	8.7
**800***	600	**0,60**	816	**80**	23.6	11.6

* 1er et 2ième paliers

	English (GB)	German (D)	Danish (DK)	Spanish (E)	French (F)	Italian (I)
	Single Use	Zum Einmalgebrauch	Til engangsbrug	De un sólo uso	Usage unique	Monouso
	Attention, see instructions for use	Achtung, bitte Gebrauchsanweisung beachten	Se brugsanvisning	Atención, ver instrucciones de uso	Attention, voir notice d'utilisation	Attenzione vedere l'istruzioni per l'uso
STERILE EO	Sterile using ethylene oxide	Sterilisiert mit Ethylenoxid	Ethylenoxid steriliseret	Esterilizado, por óxido de etileno	Stérile, utilisant l'oxyde d'éthylène	Sterilizzato ad eto
LOT	Batch Code	Charger Nummer	Batch nummer	Número de lote	Numéro de lot	Lotto no.
	Date of Manufacture	Herstellungs datum	Fremstillings dato	Fecha de fabricación	Date de fabrication	Data di produzione
	Use by	Zu verwenden bis	Anvendes før	Fecha de caducidad	A utiliser avant	Scadenza
	Keep away from heat	Vor Wärme schützen	Må ikke opbevares varmt	Manter alejado del calor	A garder à l'abri de la chaleur	Tenere lontano rae fonti di calore
	Keep dry	Trocken aufbewahren	Opbevares tørt	Guardar en lugar seco	Garder au sec	Tenere in luogo asciutto
REF	Catalogue number	Bestellnummer	Katalog nummer	Número de referencia	Code produit	Nr. Cat.
Mentions	Single patient use. Sterile unless unit container is opened or damaged. Destroy after single patient use. Do not re-sterilise	Zum Gebrauch bei einem Patienten. Nur bei ungeöffneter oder unbeschädigter Verpackung steril. Nach einmaligem Gebrauch vernichten. Nicht erneut sterilisieren.	Til brug på én patient. Sterile medmindre pakken er åbnet eller beskadiget. Skal destrueres efter brug på én patient. Må ikke resteriliseres.	Para uso en un solo paciente. Estéril a menos que el envase esté abierto o estropeado. Destruir después del uso en un solo paciente. No volver a esterilizar.	Usage patient unique. Stérile sauf si l'emballage est ouvert ou endommagé. A détruire après usage patient unique. Ne pas restériliser.	Monopaziente. Sterile purché la confezione rimanga integra e in perfette condizioni. Distruggere dopo l'uso monopaziente. Non risterilizzare.

6/6

17.5. ANNEXE E : DECLARATION DE CONFORMITE DES VENTOUSES OBSTETRICALES ICUP

COLLIN

DECLARATION CE DE CONFORMITE

Je soussigné, Monsieur Michel SIRAMY, Responsable Qualité & Affaires Réglementaires, dans la société COLLIN, 3 rue de Robinson, 92227 BAGNEUX, assure et déclare que le dispositif médical :

Ventouse obstétricale stérile à usage unique « iCup »
Référence : **JL 7000**

Répond aux exigences essentielles et aux recommandations de la Directive Européenne 93/42/CE.

Est en conformité avec l'arrêté du 20 avril 2006 du Ministère de la Santé et des Solidarités relatif aux dispositifs médicaux.

Cette déclaration est faite sur la base du Certificat **N° 14459** relatif au marquage CE selon la Directive Européenne 93/42/CE – **Annexe II** établi par le **LNE / G-MED**, 1 rue Gaston Boissier 75724 Paris, organisme notifié n° 0459.

Fait à Bagneux, le 17 février 2009

Michel SIRAMY

COLLIN
3, rue de Robinson
92227 Bagneux Cedex
Tél. 01 49 08 08 88
Fax 01 49 08 08 89
E-mail : info@collinmedical.fr

Siège social :
3, rue de Robinson
92227 Bagneux Cedex
France

Service clients : + 33 1 49 08 08 88
Télécopie : + 33 1 49 08 08 89
Service export : + 33 1 49 08 08 80
Service technique : + 33 1 49 08 09 28
info@collinmedical.fr
www.collinmedical.fr

COLLIN
Société anonyme
au capital de 557 640 euros
RCS Nanterre B 428 955 520
Siret 428 955 520 00039
APE 514 N
Code TVA : FR 67 428 955 520

G-MED ISO 13485

snitem

17.6. ANNEXE F : ATTESTATION CE ICUP

LNE
Le progrès, une passion à partager
Certification
Médical-Santé

ATTESTATION/ *CERTIFICATE* **N° 14459 Révision 0**
Délivrée à Paris le 22 septembre 2008
Issued in Paris on September 22nd, 2008

ATTESTATION CE / *EC CERTIFICATE*

Approbation du Système Complet d'Assurance Qualité / *Approval of full Quality Assurance System*
ANNEXE II point 3 Directive 93/42/CEE relative aux dispositifs médicaux
ANNEX II section 3 DIRECTIVE 93/42/EEC concerning medical devices

Fabricant (nom et adresse) / *Manufacturer (name and address)*

COLLIN ORL
3 rue de Robinson,
92227 BAGNEUX CEDEX FRA

Catégorie du(des) dispositif(s) / *Device(s) category*

Ventouse obstétricale stérile à usage unique

Sterile obstetric cup in single use

Le LNE/G-MED atteste qu'à l'examen des résultats figurant dans le rapport référencé H091142, le système d'assurance qualité - pour la conception, la production et le contrôle final - des dispositifs médicaux énumérés ci-dessus est conforme aux exigences de l'annexe II point 3 de la Directive 93/42/CEE.

LNE/G-MED certifies that, on the basis of the results contained in the file referenced H091142, the quality system - for design, manufacturing, and final inspection - of medical devices listed here aboved complies with the requirements of the Directive 93/42/EEC, annex II section 3

Début de validité / *Effective date :* **September 22nd, 2008 (included)**
Valable jusqu'au / *Expiry date :* **July 15th, 2010 (included)**

LNE/G-MED	CE	0459

For the General Director
Laurence DAGALLIER
Business Development and Certification Director

V0 04-07-2007
LNE - 14459 rev. 0

Laboratoire national de métrologie et d'essais • Établissement public à caractère industriel et commercial
LNE/G-MED • Organisme notifié n° 0459
1, rue Gaston Boissier - 75724 Paris Cedex 15 • Tél. : 01 40 43 37 00 • Fax : 01 40 43 37 37 • www.lne.fr • www.gmed.fr

17.7. ANNEXE G : TABLEAU D'IDENTIFICATION DES INVESTIGATEURS REFERENTS

Prénom, Nom	Adresse professionnelle
Dr Véronique Equy en remplacement du Pr Jean-Patrick Schaal	Clinique Universitaire de Gynécologie Obstétrique, pôle Couple - Enfant, CHU de Grenoble
Didier Riethmuller	Service de Gynécologie Obstétrique, CHU de Besançon
Michel Dreyfus	Service de Gynécologie Obstétrique, CHU de Caen
Bruno Langer	Service de Gynécologie Obstétrique, CHU de Strasbourg
Françoise Vendittelli	Service de Gynécologie Obstétrique, CHU de Clermont-Ferrand
Victoire Cabaud	Service de Gynécologie Obstétrique, CHG de Chambéry

17.8. ANNEXE H : CV DES INVESTIGATEURS REFERENTS

CURRICULUM VITAE ABREGE

Nom : Schaal

Prénom : Jean-Patrick

Adresse professionnelle : Gynécologie Obstétrique - CHU Grenoble – BP 217 – 38043 Grenoble Cedex 09

Téléphone : 04 76 76 94 50

N° d'inscription au Conseil de l'ordre des médecins : 8243

Email : JPSchaal@chu-grenoble.fr

Titres et diplômes universitaires

Professeur des Universités - Praticien Hospitalier

MD PhD

Habilitation à Diriger les Recherches

Fonctions

Chef de département gynécologie obstétrique CHU Grenoble

Synthèse des publications

SCHAAL JP, RIETHMULLER D, BERTHET J
Manœuvre instrumentales et manuelles lors de l'accouchement.
Rev Prat, 1999, 49 (2) : 139-45.

SCHAAL JP, MAILLET R, RIETHMULLER D
Accouchement, délivrance et suites de couches normales.
Rev Prat, 2004, 54 (9) : 1025-35.

SCHAAL JP, RIETHMULLER D, MENGET A
Extractions instrumentales du fœtus : ventouse obstétricale.
Encycl Méd Chir (Elsevier, Paris), Obstétrique, 2004 Pp 156-79.

MAISONNETTE-ESCOT Y, RIETHMULLER D, CHEVRIÈRE S, BECHER P, FLORET N, ZURLINDEN B, TESSIER G, MAILLET R, SCHAAL JP
L'extraction instrumentale par spatules de Thierry : étude de la morbidité maternofœtale.
Gynecol Obstet Fertil, 2005, 33 (4) : 208-12.

SCHAAL JP, RIETHMULLER D, MAILLET R, UZAN M
Mécanique et Techniques Obstétricales. 3ème édition
Sauramps Médical Ed., Montpellier, 2007, 922 p.

Date : 24/02/09

Signature :

CURRICULUM VITAE ABREGE

Nom : Riethmuller

Prénom : Didier

Adresse professionnelle : Service de Gynécologie Obstétrique - CHU Saint Jacques – 25000 Besançon

Téléphone : 03 81 21 86 64

N° d'inscription au Conseil de l'ordre des médecins : 25/3145

Email : didier.riethmuller@univ-fcomte.fr

Titres et diplômes universitaires

Professeur des Universités - Praticien Hospitalier

MD PhD

Habilitation à Diriger les Recherches

Fonctions

Médecin Adjoint du pôle Mère-Femme

Synthèse des publications

Broche DE, Ramanah R, Collin A, Mangin M, Vidal C, Maillet R, **Riethmuller D.** [Term-breech presentation: predictive factors of cesarean section for vaginal-birth failure]. J Gynecol Obstet Biol Reprod 2008;37:483-92.

Collin A, Dellis X, Ramanah R, Courtois L, Sautière JL, Martin A, Maillet R, **Riethmuller D.** [Severe shoulder dystocia: study of 14 cases treated by Jacquemier's maneuver]. J Gynecol Obstet Biol Reprod 2008;37:283-90.

Courtois L, Becher P, Maticot-Baptista D, Cour A, Zurlinden B, Millet P, Maisonnette-Escot Y, **Riethmuller D**, Maillet R. [Instrumental extractions using Thierry's spatulas: evaluation of the risk of perineal laceration according to occiput position in operative deliveries]. J Gynecol Obstet Biol Reprod 2008;37:276-82.

Broche DE, **Riethmuller D**, Maticot-Baptista D, Ramanah R, Cossa S, Maillet R. [Assisted vaginal delivery using the vacuum extractor in frank breech presentation]. J Gynecol Obstet Biol Reprod 2008;37:143-8.

16/02/2009

CURRICULUM VITAE (*) abrégé des investigateurs

Nom : DREYFUS
Prénom : Michel
Adresse professionnelle : Gynécologie-Obstétrique et Médecine de la Reproduction– CHRU Boulevard Clémenceau 14033 Caen Cedex
Téléphone : 02.31.27.25.33

N° d'inscription au Conseil de l'ordre des médecins : 3951/14
Email : dreyfus-m@chu-caen.fr

Titres et diplômes universitaires

Diplôme d'état de Docteur en Médecine (Thèse, Strasbourg 1984). Prélèvements précoces de villosités choriales. Techniques et perspectives d'avenir.

Certificat Universitaire d'Echographie Gynécologique et Obstétricale (Strasbourg 1987).

Certificat d'Etudes Statistiques Appliquées à la Médecine (Paris, 1992).

Diplôme d'Etudes Approfondies de Sciences Chirurgicales, option cancérologie Université Pierre et Marie Curie, Paris VI, 1992.

Fonctions

PU PH depuis 1999 au CHRU de Caen
Coordinateur du réseau bas normand de périnatalité

Synthèse des publications

1. **Dreyfus M**, Hedelin G, Kutnahorsky R, Lehmann M, Viville B, Langer B, Fleury A, M'Barek M, Treisser A, Wiesel ML, Pasquali JL. Antiphospholipid antibodies and preeclampsia. A case-control study. Obstet Gynecol 2001 ;97 :29-34
2. Baume S, Cheret A, Creveuil C, Vardon D, Herlicoviez M, **Dreyfus M.** Complications des accouchements assistés par ventouse. J Gynecol Obstet Biol Reprod 2004 ;33 :304-11
3. Girard B, Beucher G, Muris C, Simonet T, **Dreyfus M.** Sulfate de magnésium et pré-éclampsie sévère. Innocuité en pratique courante dans des indications ciblées. J Gynecol Obstet Biol Reprod 2005 ;34 :17-22
4. Denoual-Ziad C, Hors Y, Delande I, Lezin B, Herlicoviez M, **Dreyfus M.** Comparaison de l'efficacité entre dispositif intravaginal et gel vaginal de dinoprostone dans la maturation cervicale à terme en pratique quotidienne. J Gynecol Obstet Biol Reprod 2005 ;34 :62-8
5. Muris C, Girard B, Creveuil C, Durin L, Herlicoviez M, **Dreyfus M.** Management of premature rupture of membranes before 25 weeks.Eur J Obstet Gynecol Reprod Biol. 2007;131:163-8
6. Grossetti E, Vardon D, Creveuil C, Herlicoviez M, **Dreyfus M.** Rupture of the scarred uterus. Acta Obstet Gynecol Scand. 2007;86:572-8.

Caen, le 17/12/09

CURRICULUM VITAE ABREGE

Nom : LANGER

Prénom : Bruno

Adresse professionnelle : Département de Gynécologie-Obstétrique, Hôpital de Hautepierre, CHU de STRASBOURG 67098 cedex

Téléphone : 03 88 12 75 02

N° d'inscription au Conseil de l'ordre des médecins : 4473

Email : Bruno.Langer@chru-strasbourg.fr ou b.langer@mac.com

Titres et diplômes universitaires

Professeur des Universités-Praticien Hospitalier

Fonctions

Responsable du secteur Obstétrique du Pôle de Gynécologie-Obstétrique du CHU de Strasbourg

Expérience en recherche clinique :

une publication OU **une participation au moins à une étude en recherche clinique** OU **un diplôme en recherche clinique**.

Multicenter study on the clinical value of fetal pulse oximetry.
II. Compared predictive values of pulse oximetry and fetal blood analysis.
Carbonne B., **Langer B.**, Goffinet F., Audibert F., Tardif D., Le Goueff F., Laville M., Maillard F., and French Study Group on Fetal Pulse Oximetry.
Am J Obstet Gynecol 1997;177:593-8.
Second and third medical termination of pregnancy with misoprostol without mifepristone. Langer B., Peter C, Firtion C, David E, Haberstich R.Fetal Diag Ther 2004 ; 19 : 266-70
A French randomized controlled trial of ST analysis in a population with abnormal CTG during labor.
Vayssiere Ch., David E., Meyer N., Haberstich R., Sebahoun V., Roth E., Favre R., Nisand I., Langer B.
Am J Obstet Gynecol 2007 ; 197 : 299.e1-299.e6.

le 20/03/2009

Prof. Bruno LANGER
Département de Gynécologie-Obstétrique
Hôpital de Hautepierre
67098 Strasbourg cedex
Tél. 03 88 12 75 02

1

CURRICULUM VITAE (*) abrégé des investigateurs

Nom : VENDITTELLI
Prénom : Françoise
Adresse professionnelle : Maternité de l'hôtel Dieu, CHU de Clermont-Ferrand. Boulevard Léon Malfreyt, 63058 Clermont-Ferrand, cedex 1.
Téléphone : 04 73 75 05 70
N° d'inscription au conseil de l'ordre des médecins : 63/ 5168
Email : fvendittelli@chu-clermont-ferrand.fr

Titres et diplômes universitaires

MD PhD
Habilitation à Diriger les Recherches
Ancien chef de clinique assistant des hôpitaux

Fonctions

PH en Gynécologie Obstétrique
PH coordinateur du Réseau de Santé Périnatale d'Auvergne

Synthèse des publications

Chazeron ID, Llorca PM, Ughetto S, **Vendittelli F**, Boussiron D, Sapin V, Coudore F, Lémery D. Is pregnancy the time to change alcohol consumption Habits in France? Alcohol Clin Exp Res 2008; 32:868-73.

Vendittelli F, Olivier Rivière; Crenn-Hébert C, M; Rozan MA, Maria B, Jacquetin B and the obstetricians of the AUDIPOG sentinel network Is a breech presentation at term more frequent in women with a history of cesarean delivery? Am J Obstet Gynecol, 2008; 198:521-6.

Vendittelli F, Pons JC, Lemery D, Mamelle N. The term breech presentation: neonatal results and obstetric practices in France. Eur J Obstet Gynecol Reprod Biol 2006 ;125 :176-184

Laurichesse Delmas H, Winer N, Gallot D, Lopes K, Perrotin F, Fluncker S, Geissler F, Beaufrere AM, **Vendittelli F**, Coutures C, Lémery D. Prenatal diagnosis of thrombosis of the dural sinuses: report of six cases, review of the literature and suggested management. Ultrasoun Obstet Gynecol 2008; 32:188-98.

Chazeron ID, Llorca PM, Ughetto S, Coudore F, Boussiron D, Perriot J, **Vendittelli F**, Sapin V, Lémery D. Occult exposure to environmental tobacco smoke exposure. Tobacco Control 2007; 16:64-5.

Le 16 février 2009

Dr VENDITTELLI Françoise
Praticien Hospitalier
Maternité Hôtel-Dieu

CURRICULUM VITAE (*) abrégé des investigateurs

Nom : CABAUD
Prénom : Victoire
Adresse professionnelle :
Téléphone : 04 79 68 40 17
Email : victoire.cabaud@ch-chambery.fr
N° d'inscription au conseil de l'ordre des médecins : 73102354 5

Titres et diplômes universitaires

MD
Interne en chirurgie des Hôpitaux de l'APHP.
Obtention du DES de gynécologie-obstétrique le 31 octobre 1995.
Chef de Clinique assistant à la Pitié Salpetrière dans le service de Gynécologie-Obstétrique du Professeur DARBOIS pendant 2 ans à partir de novembre 1995.
Praticien hospitalier à temps plein au Centre Hospitalier de Chambéry dans le service de Gynécologie-Obstétrique depuis mars 1998.

Fonctions

PH en Gynécologie Obstétrique chef de service au CHG de Chambéry

Participation Etude Micado

Chambéry, le 2/3/09

[illegible]

17.9. ANNEXE I : LISTE DES INVESTIGATEURS

LIEU	NOM	PRENOM	FONCTION	N° CNOM	N°ADELI
CHU Grenoble	Pr SCHAAL	Jean Patrick	Chef de département gynécologie obstétrique	**38 8243**	**38 10 8243 7**
CHU Grenoble	Pr DEBILLON	Thierry	Responsable de la clinique universitaire de médecine néonatale et réanimation pédiatrique	**38 8069**	
CHU Grenoble	Dr EQUY	Véronique	PH - gynéco obstétrique	**38 7792**	**38 10 7792 4**
CHU Grenoble	Dr ROSIER	Patrick	PH - gynéco obstétrique	**38 4267**	**38 10 4267 0**
CHU Grenoble	Dr TOURNADE	Danielle	PH - gynéco obstétrique	**38 7700**	**38 10 7700 7**
CHU Grenoble	Dr PLONKA	Sophie	Chef de clinique - gynéco obstétrique	**38 9015**	**38 10 9015 8**
CHU Grenoble	Dr HOFFMANN	Pascale	PH - gynéco obstétrique	**38 7325**	**38 10 7325 3**
CHU Grenoble	Dr PETITPERRIN	Fabien	Chef de clinique - gynéco obstétrique	**38 8861**	**38 10 8861 6**
CHU Grenoble	Dr BECHERRAWY	Céline	Assistant chef de clinique - gynéco obstétrique	**38 9056**	**38 10 9056 2**
CHU ST Jacques	Pr RIETHMULLER	Didier	Médecin adjoint du pôle Mère-Femme gynécologue obstétricien	**25 3145**	
CHU ST Jacques	Dr COLLIN	Arnauld	Assistant chef de clinique	**25 4440**	
CHU ST Jacques	Dr MAISONNETTE ESCOT	Yolande	PH gynécologie obstétrique	**25 3949**	
CHU ST Jacques	Dr SAUTIERE	Jean Loup	PH gynécologue obstétricien	**25 2729**	
CHU ST Jacques	Dr COSSA	Stéphane	PH gynécologue obstétricien	**25 4137**	
CHU ST Jacques	Dr RAMANAH	Rajeev	Assistant chef de clinique	**25 4213**	**25 10 4213 1**
CHU ST Jacques	Dr COURTOIS	Laurent	Assistant chef de clinique	**25 4314**	
CHRU Caen	Pr DREYFUS	Michel	PU PH / coordinateur du réseau bas normand de périnatalité	**14 3951**	
CHRU Caen	Dr EDOUE	Cyril	Chef de clinique gynéco obstétrique	**14 4888**	
CHRU Caen	Dr BEUCHER	Gaël	PH - gynécologie obstétrique	**14 4851**	
CHRU Caen	Dr MURIS	Catherine	Assistant chef de clinique	**14 4925**	
CHRU Caen	Dr CHERET BENOIST	Angélique	PH - gynécologie obstétrique	**14 4228**	
CHRU Caen	Dr VARDON	Delphine	PH responsable de la salle d'accouchement	**14 4096**	
CHRU Caen	Dr BENOIST	Guillaume	Chef de clinique	**14 5009**	**14 10 5009 6**
CHRU Caen	Dr DENOUAL ZIAD	Christine	PH en gynécologie obstétrique	**10 2134**	**10 00 2134 1**
CHRU Caen	Dr LEFEVRE	Paul	assistant spécialiste	**14 4894**	**14 10 4894 2**
CHU Clermont Ferrand	Dr VENDITTELLI	Françoise	PH en gynécologie obstétrique	**63 5168**	
CHU Clermont Ferrand	Pr LEMERY	Didier	PU PH gynécologue obstétricien		**10003162251**
CHU Clermont Ferrand	Pr GALLOT	Denis	PU PH gynécologue obstétricien	**63 4764**	
CHU Clermont Ferrand	Dr AMBLARD	Joël	PH en gynécologie obstétrique	**63 4853**	

CHU Clermont Ferrand	Dr ACCOCEBERY	Marie	Assistant chef de clinique	**63 5437**	**63 10 5437 6**
CHU Clermont Ferrand	Dr VELEMIR	Luka	Assistant chef de clinique	**63 5586**	
CHU Clermont Ferrand	Dr NIRO	Julien	Assistant chef de clinique	**63 5696**	
CHU Clermont Ferrand	Dr SAVARY	Denis	PH en gynécologie obstétrique	**63 5305**	
Hopital de Hautepierre	Pr LANGER	Bruno	PU PH en gynéco obstétrique	**67 4473**	
Hopital de Hautepierre	Dr BOUDIER	Eric	PH en gynécologie obstétrique	**67 6409**	
Hopital de Hautepierre	Dr THOMA	Véronique	PH en gynécologie obstétrique	**67 8974**	
Hopital de Hautepierre	Dr FRITZ	Gabrielle	PH en gynécologie obstétrique	**67 9263**	
Hopital de Hautepierre	Dr PERRICARD	Bertrand	Assistant chef de clinique	**67 9590**	
Hopital de Hautepierre	Dr PRADOS	Eric	Assistant chef de clinique	**67 9297**	
Hopital de Hautepierre	Dr YOUSSEF AZER	Cherif	PH en gynécologie obstétrique	**67 9205**	**67 10 9205 4**
Hopital de Hautepierre	Dr HELMLINGER	Christine	PH en gynécologie obstétrique	**67 9196**	
Hopital de Hautepierre	Dr RAULT	Sébastien	Chef de clinique associé	**67 9768**	
Hopital de Hautepierre	Dr VIVILLE	Brigitte	PH en gynécologie obstétrique	**67 7407**	
Hopital de Hautepierre	Dr BAULON	Emmanuelle	PH en gynécologie obstétrique	**67 8940**	
Hopital de Hautepierre	Dr LOUSSERT		Chef de clinique	**67 9788**	
Hopital de Hautepierre	Dr COURDIER	Sébastien	Chef de Clinique	**67 9507**	
Hopital de Hautepierre	Dr TORBEY	Antoine	Attaché au service de gynécologie	**67 4106**	
Hopital de Hautepierre	Dr VOLTZENLOGEL	René	Attaché des hôpitaux de Strasbourg	**67 4530**	**67 10 45 30 0**
CH Chambéry	Dr CABAUD	Victoire	PH en gynécologie obstétrique	**73 2354**	**73 10 2354 5**
CH Chambéry	Dr DECROISETTE	Emmanuel	PH en gynécologie obstétrique	**73 3136**	
CH Chambéry	Dr BOULET	Stéphanie	Médecin assistant en gynécologie obstétrique		**73 10 30 409**

17.10. ANNEXE J : DELEGATIONS DE TACHES

Instituts thématiques Inserm
Institut national de la santé et de la recherche médicale

CHU Grenoble

Fiche de délégation de tâches

Etude : ICUP **Promoteur :** CHU Grenoble **Centre :** CAEN

Je soussigné(e) Dr……………………., médecin investigateur de l'étude, autorise les personnes suivantes à réaliser les tâches spécifiques indiquées dans le cadre de cette étude.

Nom Prénom Signature	Initiales	Rôle dans l'étude (indiquer les codes correspondants*)	Date de début	Date de fin
BEUCHER GAEL	GB	1, 2, 3, 4	__/__/____	__/__/____
EBOUÉ CYRIL	EC	1, 2, 3, 4	__/__/____	__/__/____
MURIS Catherine	CM	1, 2, 3, 4	__/__/____	__/__/____
VARDON Delphine	DV	1, 2, 3, 4	__/__/____	__/__/____
BENOIST Guillaume	GB	1, 2, 3, 4	__/__/____	__/__/____

*** : rôle dans l'étude :**

1. information et recueil du consentement (uniquement un médecin inscrit à l'ordre et justifiant d'une expérience dans le domaine médical étudié)
2. Prescription médicamenteuse et biologique
3. Consultations et examens médicaux
4. Remplissage du CRF
5. Gestion des prélèvements biologiques
6. Recueil des échantillons biologiques
7. Autre (précisez) :

Fait à Caen, le 19/2/09 Signature

Doc Ass : Délég tâches V2.0 Page …./….

Instituts thématiques Inserm
Institut national de la santé et de la recherche médicale

CHU Grenoble

Fiche de délégation de tâches

Etude : ICUP **Promoteur :** CHU Grenoble **Centre :** CAEN

Je soussigné(e) Dr……………………., médecin investigateur de l'étude, autorise les personnes suivantes à réaliser les tâches spécifiques indiquées dans le cadre de cette étude.

Nom Prénom Signature	Initiales	Rôle dans l'étude (indiquer les codes correspondants*)	Date de début	Date de fin
DENOUAL-ZIAD Christine	CDZ	1, 2, 3, 4	__/__/____	__/__/____
CHERET-BENOIST Angélique	ACB	1, 2, 3, 4	__/__/____	__/__/____
LEFEVRE Paul	PL	1, 2, 3, 4	__/__/____	__/__/____
			__/__/____	__/__/____
			__/__/____	__/__/____

*** : rôle dans l'étude :**

1. information et recueil du consentement (uniquement un médecin inscrit à l'ordre et justifiant d'une expérience dans le domaine médical étudié)
2. Prescription médicamenteuse et biologique
3. Consultations et examens médicaux
4. Remplissage du CRF
5. Gestion des prélèvements biologiques
6. Recueil des échantillons biologiques
7. Autre (précisez) :

Fait à Caen, le 19/2/09 Signature

Doc Ass : Délég tâches V2.0 Page …./….

Instituts thématiques Inserm
Institut national de la santé et de la recherche médicale

CHU

Fiche de délégation de tâches

Etude : ICUP **Promoteur : CHU de GRENOBLE** **Centre : CHU CHAMBERY**

Je soussigné(e) Dr. Cabaud……, médecin investigateur de l'étude, autorise les personnes suivantes à réaliser les tâches spécifiques indiquées dans le cadre de cette étude.

Nom Prénom<br>Signature	Initiales	Rôle dans l'étude (indiquer les codes correspondants*)	Date de début	Date de fin
DEBROISETTE Emmanuel	ED	1 4	__/__/____	__/__/____
BOULET Stéphanie	BS	1 4	__/__/____	__/__/____
			__/__/____	__/__/____
			__/__/____	__/__/____
			__/__/____	__/__/____

*** : rôle dans l'étude :**

1. information et recueil du consentement (uniquement un médecin inscrit à l'ordre et justifiant d'une expérience dans le domaine médical étudié)
2. Prescription médicamenteuse et biologique
3. Consultations et examens médicaux
4. Remplissage du CRF
5. Gestion des prélèvements biologiques
6. Recueil des échantillons biologiques
7. Autre (précisez) :

Fait à Chambéry, le …2/3/09……………… Signature

Délég tâches ICUP / CHU CHAMBERY Page …./….

Instituts thématiques Inserm
Institut national de la santé et de la recherche médicale

CHU

Fiche de délégation de tâches

Etude : ICUP **Promoteur : CHU de GRENOBLE** **Centre : CHU CLERMONT FERRAND**

Je soussigné(e) Dr. Jacquetin……., médecin investigateur de l'étude, autorise les personnes suivantes à réaliser les tâches spécifiques indiquées dans le cadre de cette étude.

Nom Prénom<br>Signature	Initiales	Rôle dans l'étude (indiquer les codes correspondants*)	Date de début	Date de fin
VELEMIR Luka	L.V	1,3,4,7	__/__/____	__/__/____
NIRO Julien	NJ	1,3,4,7	__/__/____	__/__/____
SAVARY Denis	SD	1,3,4,7	__/__/____	__/__/____
LEMERY Didier	LD	1,3,4,7	__/__/____	__/__/____
ACCOCEBERRY Marie<br>GALLOT Denis	AM<br>GD	1,3,4,7<br>1,3,4,7	__/__/____	__/__/____
AMBLARD Joël	AJ	1,3,4,7		

*** : rôle dans l'étude :**

1. information et recueil du consentement (uniquement un médecin inscrit à l'ordre et justifiant d'une expérience dans le domaine médical étudié)
2. Prescription médicamenteuse et biologique
3. Consultations et examens médicaux
4. Remplissage du CRF
5. Gestion des prélèvements biologiques
6. Recueil des échantillons biologiques
7. Autre (précisez) : pour ventouse.

Fait à Clermont Ferrand, le …24/02/09……… Signature

Pr. B. JACQUETIN
Chef de Service
Maternité Hôtel-Dieu
CHU Clermont-Ferrand

Délég tâches ICUP / CHU CLERMONT FERRAND Page …./….

Instituts thématiques Inserm
Institut national de la santé et de la recherche médicale

CIC Grenoble — CHU Grenoble

Fiche de délégation de tâches

Etude : Venture ICup **Promoteur :** D. Riethmuller **Centre :** Besançon

Je soussigné(e) Dr Riethmuller, médecin investigateur de l'étude, autorise les personnes suivantes à réaliser les tâches spécifiques indiquées dans le cadre de cette étude.

Nom Prénom<br>Signature	Initiales	Rôle dans l'étude (indiquer les codes correspondants*)	Date de début	Date de fin
Mauvonnette Esiot Yolande	YME	1-2-3-4	__/__/____	__/__/____
SAUTIERE Jean-Loup	JLS	1.2.3.4	__/__/____	__/__/____
COSSA Stephane	SC	1-2-3-4.	__/__/____	__/__/____
COLLIN ARNAUD	AC	1-2-3-4	__/__/____	__/__/____
RAMANAH Rajeev	RR	1-2-3-4	__/__/____	__/__/____
COURTOIS Laure	LC	1-2-3-4	__/__/____	__/__/____

*** : rôle dans l'étude :**
1. information et recueil du consentement (uniquement un médecin inscrit à l'ordre et justifiant d'une expérience dans le domaine médical étudié)
2. Prescription médicamenteuse et biologique
3. Consultations et examens médicaux
4. Remplissage du CRF
5. Gestion des prélèvements biologiques
6. Recueil des échantillons biologiques
7. Autre (précisez) :

Fait à Besançon, le 28/01/09

Signature

Doc Ass : Délég tâches V2.0 Page ..../....

Instituts thématiques Inserm
Institut national de la santé et de la recherche médicale

CIC — CHU Grenoble

Fiche de délégation de tâches

Etude : ICUP **Promoteur : CHU de GRENOBLE** **Centre : CHU STRASBOURG**

Je soussigné(e) Dr. B. LANGER médecin investigateur de l'étude, autorise les personnes suivantes à réaliser les tâches spécifiques indiquées dans le cadre de cette étude.

Nom Prénom<br>Signature	Initiales	Rôle dans l'étude (indiquer les codes correspondants*)	Date de début	Date de fin
Dr B PERRICARD	BP	1+2+3+4	__/__/____	__/__/____
Dr C. YOUSSEF	C.Y	1,2,3,4	__/__/____	__/__/____
Dr G. FRITZ	G.F	1+2+3+4	__/__/____	__/__/____
Dr V. THOMA	V.T	1+2+3+4	__/__/____	__/__/____
Dr L. LOUSSERT	L.L	1,2,3,4	__/__/____	__/__/____

*** : rôle dans l'étude :**
1. information et recueil du consentement (uniquement un médecin inscrit à l'ordre et justifiant d'une expérience dans le domaine médical étudié)
2. Prescription médicamenteuse et biologique
3. Consultations et examens médicaux
4. Remplissage du CRF
5. Gestion des prélèvements biologiques
6. Recueil des échantillons biologiques
7. Autre (précisez) :

Prof. Bruno LANGER
Département de Gynécologie-Obstétrique
Hôpital de Hautepierre
67098 Strasbourg cedex
Tél. 03 88 12 75 03

Fait à Strasbourg, le 12 MAR 2009

Signature

Délég tâches ICUP / CHU STRASBOURG Page 1/....

Instituts thématiques Inserm
Institut national de la santé et de la recherche médicale

CHU

Fiche de délégation de tâches

Etude : ICUP **Promoteur :** CHU de GRENOBLE **Centre :** CHU STRASBOURG

Je soussigné(e) Dr. B. LANGER, médecin investigateur de l'étude, autorise les personnes suivantes à réaliser les tâches spécifiques indiquées dans le cadre de cette étude.

Nom Prénom<br>Signature	Initiales	Rôle dans l'étude (indiquer les codes correspondants*)	Date de début	Date de fin
Dr E. BOUDIER	E.B	1, 2, 3, 4	__/__/____	__/__/____
Dr E. BAULON	E.B	1, 2, 3, 4	__/__/____	__/__/____
Dr S. COURDIER	S.C	1, 2, 3, 4	__/__/____	__/__/____
Dr S. RAULT	S.R	1, 2, 3, 4	__/__/____	__/__/____
Dr E. PRADOS	E.P	1, 2, 3, 4	__/__/____	__/__/____

*** : rôle dans l'étude :**

1. information et recueil du consentement (uniquement un médecin inscrit à l'ordre et justifiant d'une expérience dans le domaine médical étudié)
2. Prescription médicamenteuse et biologique
3. Consultations et examens médicaux
4. Remplissage du CRF
5. Gestion des prélèvements biologiques
6. Recueil des échantillons biologiques
7. Autre (précisez) :

Fait à Strasbourg, le 12 MAR 2009

Signature

Prof. Bruno LANGER
Département de Gynécologie-Obstétrique
Hôpital de Hautepierre
67098 Strasbourg cedex
Tél. 03 88 12 75 03

Délég tâches ICUP / CHU STRASBOURG Page 2/....

Instituts thématiques Inserm
Institut national de la santé et de la recherche médicale

CHU

Fiche de délégation de tâches

Etude : ICUP **Promoteur :** CHU de GRENOBLE **Centre :** CHU STRASBOURG

Je soussigné(e) Dr. B. LANGER médecin investigateur de l'étude, autorise les personnes suivantes à réaliser les tâches spécifiques indiquées dans le cadre de cette étude.

Nom Prénom<br>Signature	Initiales	Rôle dans l'étude (indiquer les codes correspondants*)	Date de début	Date de fin
Dr R. VOLTZENLOGEL	R.V	1, 2, 3, 4	__/__/____	__/__/____
Dr A. TORBEY	A.T	1, 2, 3, 4	__/__/____	__/__/____
Dr B. VIVILLE	B.V	1, 2, 3, 4	__/__/____	__/__/____
Dr C. HELMLINGER	C.H	1, 2, 3, 4	__/__/____	__/__/____
			__/__/____	__/__/____

*** : rôle dans l'étude :**

1. information et recueil du consentement (uniquement un médecin inscrit à l'ordre et justifiant d'une expérience dans le domaine médical étudié)
2. Prescription médicamenteuse et biologique
3. Consultations et examens médicaux
4. Remplissage du CRF
5. Gestion des prélèvements biologiques
6. Recueil des échantillons biologiques
7. Autre (précisez) :

Fait à Strasbourg, le 12 MAR 2009

Signature

Prof. Bruno LANGER
Département de Gynécologie-Obstétrique
Hôpital de Hautepierre
67098 Strasbourg cedex
Tél. 03 88 12 75 03

Délég tâches ICUP / CHU STRASBOURG Page 3/....

17.11. ANNEXE K : ETUDE DE L'ICUP EN LABORATOIRE

STATICE Santé	RAPPORT DE QUALIFICATION<br>N° **0208-2129-24-01**	**IMP046**<br>Indice 7

rien

Qualification concernant:	Traçabilité		A remplir si applicable	
☐ Scellage:<br>☐ Moulage, Surmoulage:<br>☑ Collage: résistance à la traction	Moyens utilisés	Capteur de traction IC 17<br>Colonne de traction	Client :	COLLIN
☐ Résistance à la traction:<br>☐ Test de fatigue:<br>☐ Etanchéité:	Composants utilisés	Tube armé Ø5F<br>Tube PVC Ø3xØ5<br>Colle Loctite 4011<br>Primaire 7701<br>Colle Loctite UV 3211	Réf. Produit:	**2129 300 24**
☐ Surmoulage:<br>☐ Résistance à la fuite:<br>☐ Autre :	Matières utilisées		Désignation:	**iCup v2**

Objectifs:
Vérifier la tenue du collage du tube armé dans le tube PVC

Protocole d'essais :

- Coller à la 4011 (+primaire) 5 tubes armés de 50mm dans 5 tubes PVC de 50mm en laissant le tube armé dépassé de 10mm
- Coller à la 3211 5 tubes armés de 50mm dans 5 tubes PVC de 50mm en laissant le tube armé dépassé de 10mm
- Réaliser des essais de traction à 200mm/min

Critères de vérification :

- Choisir la colle qui nous donne le collage le plus résistant.

**Protocole:**	Vérifié par: **AM**	Validé par:	**AM et MD**	Date:	**14/02/2008**
**Essais:**	Réalisés par: **AM**			Date:	**14/02/2008**

STATICE Santé	RAPPORT DE QUALIFICATION<br>N° **0208-2129-24-01**	**IMP046**<br>Indice 7

Résultats des essais:

Valeurs des forces de ruptures pour le collage à 4011 :
Echantillon N 1 : 25N -> rupture du collage
Echantillon N 2 : 44N -> rupture du tube armé au niveau des mors
38N -> rupture du collage
Echantillon N 3 : 31N -> rupture du collage
Echantillon N 4 : 29N -> rupture du collage
Echantillon N 5 : 44N -> rupture du tube armé au niveau des mors
43N -> rupture du tube armé au niveau des mors

Valeurs des forces de ruptures pour le collage à 3211 :
Echantillon N 1 : 25N -> rupture du collage
Echantillon N 2 : 13N -> rupture du collage
Echantillon N 3 : 6N -> rupture du collage
Echantillon N 4 : 10N -> rupture du collage
Echantillon N 5 : 17N -> rupture du collage

Conclusion:
On choisira le colle Loctite 4011 avec du primaire 7701 pour réaliser le collage.

Sensibiliser les opératrices sur le temps de maintien du collage.

Qualification approuvée: Par: Date:
☑ oui
☐ non **FG** **14/02/2008**
☐ en attente

Implications documentaires (si applicables)

Décisions suite à l'attente:

Diffusé au client ☐ oui ☑ non
si oui, date :
à :

Si qualification non approuvée:
Actions envisagées:

Voir rapport de qualif en date du :

STATICE Santé

RAPPORT DE QUALIFICATION

N° 0507-2129 11-01

IMP046

Indice 7

Résultats des essais:

I) Choix de la méthode de sertissage

assemblage du système à l'aide d'un boulon

	câble dénudé	serti à	débordement de matière	écrasement	Nombre de levé/posé	Câble cassé	Principe
méthode 1	x	50 bars		1.96	20		OK
méthode 2 (1)		50 bars	x	1.76	20		OK
méthode 2 (2)		50 bars	x	1.76	20		OK
méthode 3		6 bars	x	1.8	5	x	non OK
méthode 4	x	6 bars		1.9	8	x	non OK
méthode 5	x	6 bars		2.2	9	x	non OK
méthode 6		50 bars		1.76	5	x	non OK

=> Choix de la méthode 2 :

Enfiler une bague de sertissage à une extrémité de la cordelette de traction ;
Placer l'ensemble dans l'outil à sertissage de façon à ce que la bague soit à 1 mm du bout de la cordelette ;
Placer le poinçon sur l'outil à sertissage ;
Descendre en vitesse rapide la presse jusqu'à ce qu'il y ait contact ;
Descendre en vitesse lente avec une pression de 50 bars ;
Remonter la presse en vitesse rapide ;
Enlever la pièce obtenue.

II)Validation du sertissage

Les 10 sertissages ne présenent aucune déterioration de la bague ni de détachement du câbles;
Présence du bourlet de gaine de chaque côté de la bague sertie pour les 10 serissages.

III) Essai de traction

Les essais de traction réalisés sur les 10 cupules minicup répondent bien aux critères de vérification.

Conclusion:

- La méthode de sertissage (n 2) répond au cahier des charges et est validée.
- La tenue en traction du sertissage est validée.

Qualification approuvée: Par : SE MD Date : 24/05/2007

☑ oui
☐ non
☐ en attente

Implications documentaires (si applicables)

Décisions suite à l'attente:

Diffusé au client ☐ oui ☐ non

si oui, date :
à :

Si qualification non approuvée:

Actions envisagées:

Voir rapport de qualif en date du :

STATICE Santé	RAPPORT DE QUALIFICATION<br>N° 0607-2129 24-01	IMP046<br>Indice 7

Qualification concernant:	Traçabilité		A remplir si applicable	
☐ Scellage:<br>☐ Moulage:<br>☐ Collage:	Moyens utilisés	Outillage "ref 3129 100 12" de decoupe mousse "ref 2129 304 24".<br>Air comprimé réseau 6bars<br>Pied à coulisse IC60<br>Chrono IC52	Client :	COLLIN ORL-CMF
☐ Résistance à la traction:<br>☐ Test de fatigue:<br>☐ Etanchéité:	Composants utilisés	Cupule+bouchon+ventouse assemblée (pièces seulement mise en place et non collée) du lot 07-D (N Lot Aimée Griffond)	Réf. Produit:	2129 300 24
☐ Surmoulage:<br>☐ Résistance à la fuite:<br>☑ Autre : Découpe de la mousse	Matières utilisés	Bande de mousse découpée 62mm 1mm de largeur pour 1500mm de longueur	Désignation:	Cupule minicup

Objectifs:
Valider les performances du posage semi-automatique de découpe "mousse" ref 3129 100 12.
La perforformance étant de découper une rondelle de mousse ép6mm à un diamètre Ø56 0.5

Protocole d'essais :

Branchement sur Air comprimé du système 3129 100 12
Mise en place de la bande de mousse dans l'outil de découpe
Appui sur le bouton de mise en route
Relachement
Décalage jusqu'au témoin de la bande de mousse
Réitération du protocole 9 fois

Prélèvement de 3 pièces

Critères de vérification :

C1: Contrôle visuel de la périphérie de la rondelle --> pas de "bavure" ; "coupe franche"
C2: Contrôle dimensionnel Ø56 0.5
C3: Contrôle de mise en place et de tenue dans le dispositif cupule 2129 300 24 --> Tenue de la mousse (face à la gravité) dans son logement pendant 10secondes

**Protocole:**	Vérifié par:	**SE**	Validé par:	**SE/OP**	Date:	**06/06/2007**
**Essais:**	Réalisés par:	**SE/OP**			Date:	**06/06/2007**

STATICE Santé	RAPPORT DE QUALIFICATION<br>N° **0607-2129 24-02**	**IMP046**<br>Indice 7

Qualification concernant:	Traçabilité		A remplir si applicable	
☐ Scellage:<br>☐ Moulage, Surmoulage:<br>☑ **Collage** : Tous les collages (résistance traction et étanchéité après stérilisation)	Moyens utilisés	Posage 3129 100 13 n 1 et n 2, Posage 3129 100 14 n 1 et n 2, Masse (bidon+poids) de 12 kg, Balance IC 117, Bassine d'eau, Manomètre IC 101, Bocal d'eau de 1,5 kg, Chronomètre IC 52, plaque de verre	Client :	**COLLIN ORL-CMF**
☐ Résistance à la traction:<br>☐ Test de fatigue:<br>☐ Etanchéité:	Composants utilisés	10 cupules minicup lot n 19975 stérilisées (n de cycle 1 : ? ,n de cycle 2 : A3060607)	Réf. Produit:	**2129 300 24**
☐ Surmoulage:<br>☐ Résistance à la fuite:<br>☐ Autre :	Matières utilisées	Primer loctite 7701 lot n 6DZ8493<br>Loctite 4011 lot n 3026 100 03	Désignation:	**Cupule minicup**

Objectifs: Vérifier la tenue du collage
Après stérilisation :
- Valider le process de collage entre le bouchon et la coche en terme de résistance à la traction et d'étanchéïté ;
- Valider le process de collage entre la cupule et la ventouse en terme de résistance à la traction et d'étanchéïté ;
- Valider le process de collage entre le tube à vide et le connecteur en terme de résistance à la traction et d'étanchéïté ;
- Valider le process de collage entre le tube à vide et le bouchon en terme de résistance à la traction et d'étanchéïté ;

Protocole d'essais :

Essais réalisés sur les mêmes cupules utilisées pour le rapport de qualification n 0507-2129 24-01 après stérilisation.

- Etanchéïté en surpression : (cf. annexe 1)
Placer la cupule minicup dans le posage 3129 100 14 n 2;
Verrouiller le posage ;
Insérer le connecteur dans l'embout du manomètre ;
Pincer le tube ;
Régler le manomètre à une pression de 360 mbar 5 mbars;
Injecter de l'air dans la cupule minicup ;
Placer le posage et la cupule minicup entièrement dans la bassine d'eau (jusqu'au connecteur) ;
Observer pendant 5 secondes ;
Sortir l'ensemble du montage de la bassine ;
Déverrouiller et enlever la pièce du posage.

- Essai de traction (1) : (cf. annexe 2)
Appliquer une force de 15 N en ventousant (avec une aspiration maximale) la ventouse sur le bocal contenant de l'eau d'une masse de 1,5 kg. Suspendre l'ensemble par le connecteur pendant 30 secondes.

- Essai de traction (2) : (cf. annexe 3)
Appliquer une force de 120 N en ventousant (avec une aspiration maximale) la ventouse sur la masse de 12 kg. Suspendre l'ensemble par la cordelette de traction pendant 30 secondes en ne lachant pas brutalement la masse.

- Etanchéïté en dépression : (cf. annexe 4)
Ventouser en faisant le vide (avec une aspiration maximale), la ventouse sur une plaque de verre et plonger l'ensemble dans la bassine d'eau (jusqu'au connecteur) pendant 1 miniute.

Après le test en traction, on réalise à nouveaule test d'étanchéïté en surpression.

Critères de vérification :

- Etanchéïté en surpression :
Pas de présence de bulles (fuites) au niveau de la cordelette de traction.

- Essai de traction (1) :
Les collages aux 2 extrémités du tube à vide supportent une force de 15 N pendant 30 secondes sans que les collages se déteriorent.

**Protocole:**	Vérifié par: **SE**		Validé par: **SE**		Date:	**11/06/2007**
**Essais:**	Réalisés par: (JV)				Date:	**11/06/2007**

STATICE Santé	RAPPORT DE QUALIFICATION<br>N° 0607-2129 24-02	IMP046<br>Indice 7

- Essai de traction (2) :
Le collage entre la cupule et la ventouse supporte une force de 120 N pendant 30 secondes sans que le collage se déteriore ;
Le collage entre la cloche et le bouchon supporte également.

- Etanchéité en dépression :
Absence d'infiltrations d'eau à l'intérieur de la cupule minicup au bout de 1 minute.

- Etanchéité en surpression :
Absence de bulles (fuites) au niveau de la cordelette de traction au bout au bout de 5 secondes.

Résultats des essais:

Essais réalisés avec une pression de 360 mbars 5 mbars;
une masse de 11,7 kg ; (soit environ 117 N)
une masse de 1,501 kg ; (soit environ 15 N)

Résultats des essais réalisés sur les 10 cupules minicup :

	Etanchéité en surpression	Essai de traction (1)	Essai de traction (2)	Etanchéité en dépression	Etanchéité en surpression	plicature
cupule 1	Aucune fuite	Tenue OK	Tenue OK	Aucune fuite	Aucune fuite	
cupule 2	Aucune fuite	Tenue OK	Tenue OK	Aucune fuite	Aucune fuite	x
cupule 3	Fuite connecteur	échec	Tenue OK	Fuite connecteur	échec	x
cupule 4	Aucune fuite	Tenue OK	Tenue OK	Aucune fuite	Aucune fuite	
cupule 5	Aucune fuite	Tenue OK	Tenue OK	Aucune fuite	Aucune fuite	x
cupule 6	Fuite connecteur	Tenue OK	échec	Fuite connecteur	échec	x
cupule 7	Aucune fuite	Tenue OK	Tenue OK	Aucune fuite	Aucune fuite	
cupule 8	Aucune fuite	Tenue OK	Tenue OK	Aucune fuite	Aucune fuite	
cupule 9	Aucune fuite	Tenue OK	Tenue OK	Aucune fuite	Aucune fuite	

Remarque :
Constat d'une plicature sans obstruction du tube à vide au niveau du collage avec la cupule.

Conclusion:

- Les fuites observées au niveau du connecteur des 2 cupules minicup sont dûes aux essais de traction réalisés dans la validation des collages avant stérilisation utilisant une longueur de tube à vide (30 cm) qui est nettement inférieure à la longueur d'utilisation réelle (2m). De plus l'insertion d'un tube souple dans un connecteur dur avec une colle rapide est difficile ce qui implique une mauvaise répartition de la colle et donc une mauvaise étanchéité.
-->solution apportée : fabrication d'un mandrin permettant un meilleur positionnement du tube à vide dans le connecteur.

- La plicature n'est pas critique car il n'y a pas obstruction du tube à vide. Ce problème est résolut en définissant un positionnement de la cupule dans le sachet (cf. annexe 5).

Contôle à 100% en fabrication de l'étanchéité.

- Le processus de collage est validé : validation de l'étanchéïté et de la résistance à la traction.

Qualification approuvée: Par: SE MD Date: 11/06/2007
☑ oui
☐ non
☐ en attente

Implications documentaires (si applicables)
☐ oui
☐ non

Décisions suite à l'attente:

Diffusé au client
☐ oui
☐ non
si oui, date :
à :

Si qualification non approuvée:
Actions envisagées:

Voir rapport de qualif en date du :

STATICE Santé	RAPPORT DE QUALIFICATION<br>N° 0607-2129 24-03	IMP046<br>Indice 7

Qualification concernant:	Traçabilité		A remplir si applicable	
☑ **Scellage :** après stérilisation<br>☐ Moulage:<br>☐ Collage:<br>☐ Résistance à la traction:<br>☐ Test de fatigue:<br>☐ Etanchéité:<br>☐ Surmoulage:<br>☐ Résistance à la fuite:<br>☐ Autre :	Moyens utilisés	Machine de test en traction (Chatillon TCD 200) IC 118, Thermoscelleuse HM460 EQC 0002, Chronomètre IC 52	Client :	**COLLIN ORL-CMF**
	Composants utilisés	6 cupules minicup postiches conditionnées stérilisées n de cycle 1 : ? ,n de cycle 2 : A3060607 (rebuts marqués d'une pastille rouge), 6 sachets STTC 350*270 PCHOC 109P/PET (oliver order no : 171860*00), Eau+encre bleue	Réf. Produit:	**2129 300 24**
	Matières utilisés		Désignation:	**Cupule minicup**

Objectifs:

- Vérifier le processus de conditionnement en terme de pelabilité, de résistance au niveau de la soudure après stérilisation selon la norme NF EN 868-5.

Protocole d'essais :

Essais réalisés sur les mêmes cupules utilisées pour le rapport de qualification n 0507-2129 24-02 après stérilisation.

- Essai de résistance à la traction de la soudure :
Essais réaliser sur 3 cupules minicup (échantillons n 1 à 3);
Essais portant sur 2 éprouvettes de 15 mm de largeur découpées à l'aide d'un scalpel et d'un modèle;
Placer l'éprouvette sur la machine de test en traction et appliquer une vitesse d'avance de 200 mm/min. (cf. annexe 1)

- Essai de pelabilité :
Essais réalisés sur les mêmes cupules minicup utilisées pour les essais de résistance à la traction des soudures ;
Ouvrir la poche du côté de la flèche et vérifier l'état de la soudure réalisée.

- Essai d'étanchéïté : (Intégrité de la poche)
Essais portant sur 3 cupules minicup (échantillons n 4 à 6);
Ouvrir l'emballage du côté de la flèche permettant d'extraire la cupule minicup. Verser de l'eau contenant de l'encre dans la poche et la suspendre pendant 15 minutes. Observer les soudures pour détecter les fuites éventuelles. (cf. annexe 2)

Critères de vérification :

- Essai de résistance à la traction de la soudure :
La force de traction doit être > à 1,5 N.

- Essai de pelabilité :
La soudure doit être uniforme.

- Essai d'étanchéïté :
La soudure ne doit pas comporter de fuites de liquide au bout de 15 minutes.

**Protocole:**	Vérifié par:	**SE, AG**	Validé par:	**SE, AG**	Date:	**11/06/2007**
**Essais:**	Réalisés par:	**(JV)**			Date:	**11/06/2007**

STATICE Santé	RAPPORT DE QUALIFICATION<br>N° 0607-2129 24-03	IMP046<br>Indice 7

Résultats des essais:

Essai de résistance à la traction des soudures :
- échantillon n 1 => traction : 4 N
  traction : 3 N
- échantillon n 2 => traction : 2,3 N
  traction : 3,1 N
- échantillon n 3 => traction : 2,35 N
  traction : 3 N

Essai de pelabilité :
- échantillon n 1 => pelabilité manuelle : bonne
- échantillon n 2 => pelabilité manuelle : bonne
- échantillon n 3 => pelabilité manuelle : bonne

Essai d'étanchéïté :
- échantillon n 4 => bonne étanchéïté de la soudure
- échantillon n 5 => bonne étanchéïté de la soudure
- échantillon n 6 => bonne étanchéïté de la soudure

Conclusion:

Le processus de conditionnement en terme de pelabilité, de résistance de la soudure et d'étanchéïté selon la norme NF EN 868-5 est validé.

Qualification approuvée: Par : SE MD Date: 11/06/2007
- [x] oui
- [ ] non
- [ ] en attente

Implications documentaires (si applicables)

Décisions suite à l'attente:

Diffusé au client
- [ ] oui
- [ ] non

si oui, date :
à :

Si qualification non approuvée:
Actions envisagées:

Voir rapport de qualif en date du :

17.12. ANNEXE L : FICHE DE DECLARATION D'EVENEMENT INDESIRABLE

Essais cliniques promus par le CHUG : Fiche de déclaration d'évènement indésirable

**Déclarant**<br>Nom* : ………………………………………………… Prénom : ……………………………………………….<br>☐ Médecin ☐ Pharmacien ☐ ARC ☐ Autre : ………………………….<br>Adresse* :………………………………………………………………………………………………………..<br>Mel : ……………………………………………………………… Tel *: ………………………………………………	**A adresser à :**<br>Dr Céline Villier - Centre Régional de Pharmacovigilance<br>CVillier@chu-grenoble.fr<br>Pharmacovigilance@chu-grenoble.fr<br>Fax : 04 76 76 56 55 Tel : 04 76 76 51 45

**Nom de l'essai* :**<br><br>**N° EudraCT* :**	**N° Patient** : ……………………………………<br>Nom* : ☐___☐___☐___☐☐ … Prénom : ☐___☐___☐___☐☐<br>Sexe* : ☐ M ☐ F<br>Age ou date de naissance* : …………………………………	Antécédents :<br><br>Poids : ……………………. Taille : …………………

**Nature de l'évènement***	Date de début	Gravité **2 à 6 : Déclaration immédiate**<br>Non grave 1<br>Hospitalisation - Prolongation d'hospit. 2<br>Séquelles 3<br>Mise en jeu du pronostic vital 4<br>Décès 5<br>Anomalie congénitale 6	Intensité<br>Légère 1<br>Modérée 2<br>Sévère 3	Evolution<br>Guérison 1<br>Non rétabli 2<br>En voie de guérison 3<br>Guérison avec séquelle 4<br>Décès 5<br>Inconnue 6	Durée	Lien de causalité avec le produit<br>Oui / Non ?	Traitement symptomatique	Déclaré le

**Nom de produit ***<br>**Y compris traitements concomitants**	Voie	Posologie	Date de début	Date de fin	Indication	**Commentaires en particulier les étiologies qui ont pu être exclues**
